# Human-derived air–liquid interface cultures decipher Alzheimer’s disease–SARS-CoV-2 crosstalk in the olfactory mucosa

**DOI:** 10.1186/s12974-023-02979-4

**Published:** 2023-12-14

**Authors:** Muhammad Ali Shahbaz, Suvi Kuivanen, Riikka Lampinen, Laura Mussalo, Tomáš Hron, Táňa Závodná, Ravi Ojha, Zdeněk Krejčík, Liudmila Saveleva, Numan Ahmad Tahir, Juho Kalapudas, Anne M. Koivisto, Elina Penttilä, Heikki Löppönen, Prateek Singh, Jan Topinka, Olli Vapalahti, Sweelin Chew, Giuseppe Balistreri, Katja M. Kanninen

**Affiliations:** 1 A.I. Virtanen Institute for Molecular Sciences, University of Eastern Finland, 70210 Kuopio, Finland; 2 Department of Virology, Faculty of Medicine, University of Helsinki, 00290 Helsinki, Finland; 3 Charité-Universitätsmedizin Berlin, Corporate Member of Freie Universität Berlin and Humboldt-Universität Zu Berlin, Institute of Virology, 10117 Berlin, Germany; 4 Institute of Molecular Genetics, Czech Academy of Sciences, 142 20 Prague, Czech Republic; 5 Department of Genetic Toxicology and Epigenetics, Institute of Experimental Medicine, Czech Academy of Sciences, 142 20 Prague, Czech Republic; 6 Department of Neurology, Neuro Centre, Kuopio University Hospital, 70210 Kuopio, Finland; 7 Brain Research Unit, Department of Neurology, School of Medicine, University of Eastern Finland, 70210 Kuopio, Finland; 8 Department of Neurology and Geriatrics, Helsinki University Hospital and Neurosciences, Faculty of Medicine, University of Helsinki, 00014 Helsinki, Finland; 9 Department of Otorhinolaryngology, University of Eastern Finland and Kuopio University Hospital, 70210 Kuopio, Finland; 10 Finnadvance, 90220 Oulu, Finland; 11 The Queensland Brain Institute, University of Queensland, Brisbane, Queensland 4072 Australia

**Keywords:** COVID-19, Alzheimer’s disease, Neurological manifestations, SARS-CoV-2, Olfactory, Anosmia, Air–liquid interface, Inflammation, Immune responses

## Abstract

**Background:**

The neurological effects of the coronavirus disease of 2019 (COVID-19) raise concerns about potential long-term consequences, such as an increased risk of Alzheimer's disease (AD). Neuroinflammation and other AD-associated pathologies are also suggested to increase the risk of serious SARS-CoV-2 infection. Anosmia is a common neurological symptom reported in COVID-19 and in early AD. The olfactory mucosa (OM) is important for the perception of smell and a proposed site of viral entry to the brain. However, little is known about SARS-CoV-2 infection at the OM of individuals with AD.

**Methods:**

To address this gap, we established a 3D in vitro model of the OM from primary cells derived from cognitively healthy and AD individuals. We cultured the cells at the air–liquid interface (ALI) to study SARS-CoV-2 infection under controlled experimental conditions. Primary OM cells in ALI expressed angiotensin-converting enzyme 2 (ACE-2), neuropilin-1 (NRP-1), and several other known SARS-CoV-2 receptor and were highly vulnerable to infection. Infection was determined by secreted viral RNA content and confirmed with SARS-CoV-2 nucleocapsid protein (NP) in the infected cells by immunocytochemistry. Differential responses of healthy and AD individuals-derived OM cells to SARS-CoV-2 were determined by RNA sequencing.

**Results:**

Results indicate that cells derived from cognitively healthy donors and individuals with AD do not differ in susceptibility to infection with the wild-type SARS-CoV-2 virus. However, transcriptomic signatures in cells from individuals with AD are highly distinct. Specifically, the cells from AD patients that were infected with the virus showed increased levels of oxidative stress, desensitized inflammation and immune responses, and alterations to genes associated with olfaction. These results imply that individuals with AD may be at a greater risk of experiencing severe outcomes from the infection, potentially driven by pre-existing neuroinflammation.

**Conclusions:**

The study sheds light on the interplay between AD pathology and SARS-CoV-2 infection. Altered transcriptomic signatures in AD cells may contribute to unique symptoms and a more severe disease course, with a notable involvement of neuroinflammation. Furthermore, the research emphasizes the need for targeted interventions to enhance outcomes for AD patients with viral infection. The study is crucial to better comprehend the relationship between AD, COVID-19, and anosmia. It highlights the importance of ongoing research to develop more effective treatments for those at high risk of severe SARS-CoV-2 infection.

**Graphical Abstract:**

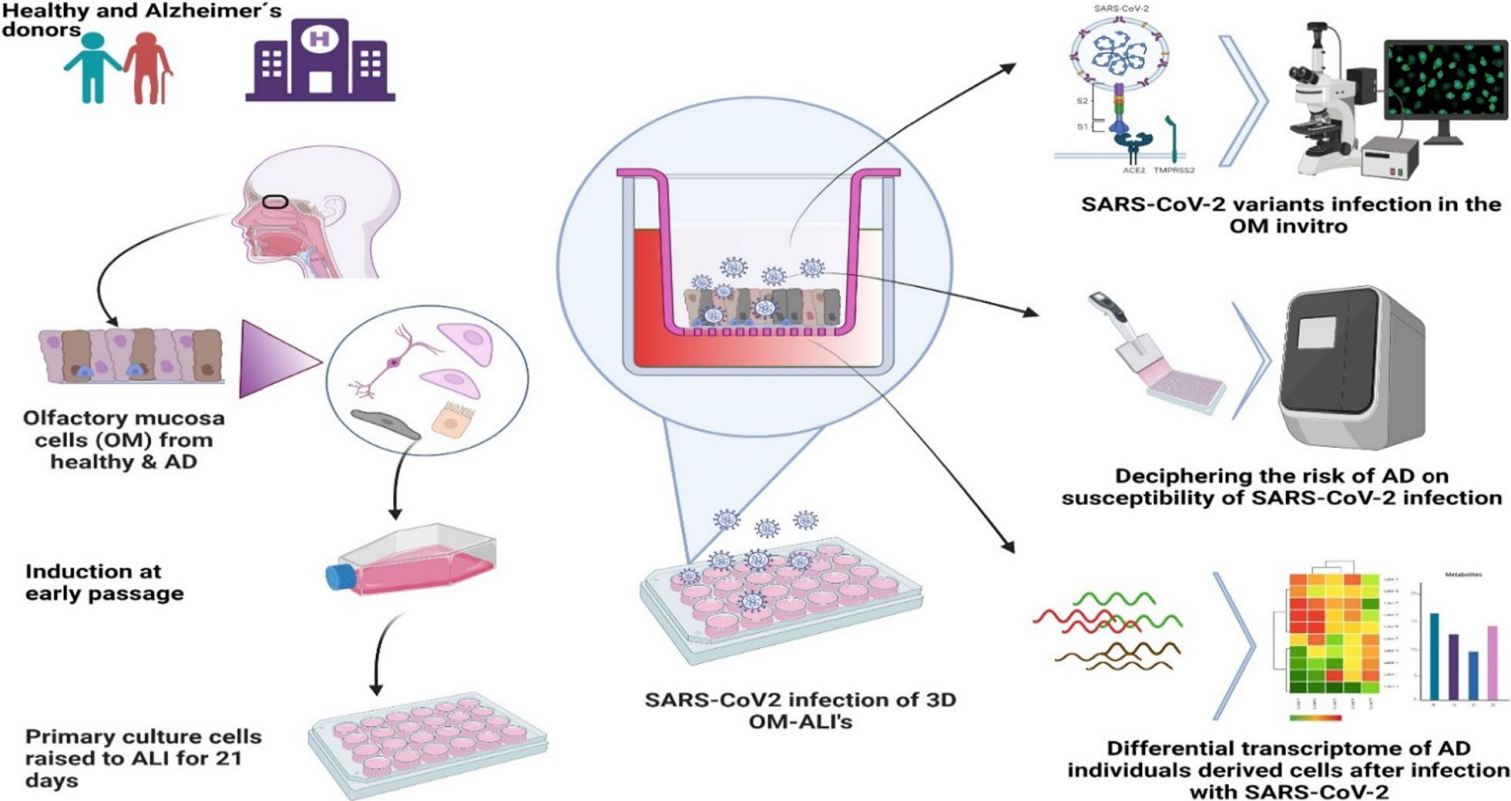

**Supplementary Information:**

The online version contains supplementary material available at 10.1186/s12974-023-02979-4.

## Introduction

Coronavirus disease (COVID-19) caused by severe acute respiratory coronavirus 2 (SARS-CoV-2) persists as a serious global health problem after three years into the pandemic. As of December 2022, there have been 652 million confirmed cases of COVID-19, including 6.7 million deaths, reported to the World Health Organization (WHO). It has been observed since the early days of the pandemic that SARS-CoV-2 predominantly attacks the human respiratory system, but also causes dysfunction in other organs including the central nervous system (CNS). A wide range of neurological symptoms has been reported to accompany the disease and affect its course. Olfactory dysfunction was recognized early in the COVID-19 pandemic [1–3] and is a strong and consistent symptom associated with SARS-CoV-2 infection [4]. Most patients show extensive or complete recovery of the ability to smell within 2–3 weeks of the first symptoms [5, 6]. However, in about 10–20% of cases, loss of the sense of smell persists for months after infection onset [6]. Furthermore, clinical evidence also indicates that a subset of patients bears long-lasting consequences (also known as long COVID) of SARS-CoV-2 infection, including olfactory dysfunction that persists even a year after the initial infection [7].

Given the CNS-related symptoms observed in COVID-19 patients, it is likely that SARS-CoV-2 can target the brain [8, 9]. A potential pathway to induce neurological manifestations occurs intranasally through the olfactory bulb via a trans-synaptic route, as supported by reported loss of olfaction in COVID-19 patients. Since the olfactory mucosa (OM) is situated at the rooftop of the nasal cavity, and directly exposed to the environment, it acts as the first line of defense against inhaled agents including viruses that could potentially enter the brain. It harbors a heterogeneous population of cells including olfactory sensory neurons (OSNs) responsible for initiating olfactory sensations, supporting cells, and cells of regenerative potential. However, evidence from research conducted since the pandemic indicates that human olfactory sensory neurons do not express or exhibit low expression of *TMPRSS2* (transmembrane protease, serine 2), and *ACE-2* (angiotensin-converting enzyme 2), two key genes involved in SARS-CoV-2 entry into the cell [10]. Instead, protein products of these two genes are abundant in samples of the whole olfactory mucosa of humans [10] and in mouse olfactory epithelium (OE) [11]. Emerging evidence suggests that in the OE, sustentacular cells which are known to support olfactory sensory neurons, express relatively high levels of ACE-2 [12, 13]. Infection of the sustentacular cells leads to dysfunction of the olfactory sensory neurons and consequentially loss of smell in COVID-infected individuals [14]. However, infection of the olfactory sensory neurons has not yet been ruled out [15, 16]. Notably, new evidence has shown other proteins such as NRP-1 (neuropilin-1), to serve as important SARS-CoV-2 receptors and thus enhance viral infectivity [17]. NRP-1 is present at high levels in the OE and in brain areas related to olfaction [18, 19]. Therefore, the olfactory pathway may constitute an important route of CNS invasion. However, the consequences of SARS-CoV-2 infection at the OM are not fully understood, partially due to a shortage of robust and effective in vitro models to study viral infection in human cells.

Alzheimer’s disease (AD) is one of the most prevalent CNS disorders associated with the comorbidity of COVID-19 [20, 21]. AD is complex and affected by age, heredity, lifestyle, and environmental factors [22]. The disease is pathologically characterized by the deposition of amyloid beta (Aβ) and neurofibrillary tangles in the brain regions responsible for memory and learning, causing a multitude of symptoms associated with dementia [23]. Interestingly, like SARS-CoV-2 infection, olfactory dysfunction is often reported in several neurodegenerative diseases [24], including AD [25–28]. Recently, Lampinen et al. 2022 showed AD-associated changes in OM cells derived from biopsies of individuals with AD [29, 30], suggesting that these cells display similar alterations seen in AD-affected brains.

The connection of AD with viral infections has been postulated for decades and is still controversial despite supporting evidence [31, 32], but little is yet known about the relationship between SARS-CoV-2 and AD [33–35]. On one hand, there appears to be an increased risk of COVID-19 infection in individuals diagnosed with AD [36], and on the other hand, long-lasting neurological consequences of SARS-CoV-2 infection may relate to the onset of AD. Furthermore, severe infection is associated with aging-related molecular features in the brain [37] and the brains of COVID-19 patients display AD-like pathological features [38]. Interestingly, interferon-induced transmembrane protein 3 gene networks are significantly enriched in AD patients and induced by several viruses, including SARS-CoV-2 [39], suggesting a potential crossroad between immune mechanisms and AD pathology. The core of the crosstalk between AD and SARS-CoV-2 could potentially be the inflammatory processes [40, 41]. However, whether COVID-19 could trigger emerging AD or accelerate its onset remains unclear. Furthermore, the effects of SARS-CoV-2 infection at the OM, a mucous membrane interacting with both the external environment and the brain, of individuals with AD have not previously been addressed.

In this study, we collected OM biopsies from cognitively healthy donors and individuals with AD to model the human OM in a 3-dimensional (3D), Air–liquid interface (ALI) culture system and investigated the effects of SARS-CoV-2 infection in the cultured cells. We explored how AD affects susceptibility to SARS-CoV-2 infection and mapped the transcriptomic landscape of SARS-CoV-2 infection both in healthy and diseased cells.

## Materials and methods

### Ethical considerations

Olfactory biopsies were obtained from the cognitively healthy and AD individuals under the approved ethical permit from the Human Research Ethics Committees (HRECs), of Northern Savo Hospital District (permit number 536/2017). Written informed consent was obtained from all subjects and proxy consent from family members of persons with mild AD dementia.

### Human olfactory mucosal biopsies

For the collection of human olfactory mucosal (OM) biopsies a relatively non-invasive procedure was carried out by an ENT surgeon at Kuopio University Hospital, Finland. A total of three cognitively healthy individuals and three individuals diagnosed with mild AD dementia participated in the study. The average age of the cognitively healthy control and AD individuals was 74.3 years and 62.3 years, respectively, representing both male and female donors. For this study, individuals diagnosed with AD had mild dementia according to Clinical Dementia Rating (CDR) and were recruited via the Brain Research Unit, Department of Neurology, University of Eastern Finland. All the individuals with AD fulfilled the NIA-AA clinical criteria of progressive AD and magnetic resonance imaging (MRI) or fluorodeoxyglucose (FDG)-positron emission tomography (PET) had showed degenerative processes, or, in Cerebrospinal fluid (CSF) biomarker examination, biomarker (Aβ, tau, and phos-tau) changes typical to AD were observed [42]. Cognitively healthy individuals were recruited via the Department of Otorhinolaryngology, Kuopio University Hospital, Finland, from donors undergoing a dacryocystorhinostomy (DCR) surgery, or from the already existing registries of the Brain Research Unit of the University of Eastern Finland. The cognition of all the study participants was evaluated utilizing the Consortium to Establish a Registry for Alzheimer’s Disease (CERAD) neuropsychological battery [43, 44].

The detailed protocol for collecting and processing OM biopsy collected from the rooftop of the nasal cavity was previously described [30, 45]. However, some modifications were made to improve the epithelial cell growth. Briefly, the tissue piece collected from the rooftop of the nasal cavity was transferred under an aseptic environment to the Biosafety Level 2 (BSL2) facility in PneumaCult ‐ Ex Plus (Stemcell Technologies) prepared according to the manufacturer’s instructions and supplemented with hydrocortisone (final concentration of 96 ng/mL) and 1% Penicillin–Streptomycin (Gibco). Processing of the tissue sample was done immediately, starting with the rinsing in cold Hank’s Balanced Salt Solution (HBSS) followed by the removal of blood and cartilage. The clean tissue sample was then enzymatically digested for 45 min with dispase II at 2.4 U/mL (Roche, Basel, Switzerland) to separate OE from lamina propria (LP). Once the OE was separated, the LP fraction was first mechanically triturated followed by treatment with PneumaCult ‐ Ex Plus media containing 0.25 mg/mL collagenase H (Sigma-Aldrich, St. Louis, MO, USA) for up to 10 min. Once the OE and LP were completely digested, both the OE and LP were combined and seeded on poly-d-lysine (Sigma-Aldrich) coated wells of a 6-well plate in supplemented PneumaCult ‐ Ex Plus media. The cultures were then incubated at 37 °C, 5% CO_2_ to allow cells to migrate out of the digested tissue pieces and proliferate. Half of the culture media was changed every 2–3 days for a total of 8–14 days before passaging the cultures and freezing the primary cell lines in liquid nitrogen for later use in a freezing media containing 90% PneumaCult‐Ex Plus Media and 10% dimethyl sulfoxide (DMSO). Cells in primary passages 2–3 were used for air–liquid interface cultures as described below.

### Establishment and maintenance of air–liquid interface culture (ALI)

Cryopreserved human primary olfactory mucosal cells were thawed and grown for 3–5 days in submerged cultures in PneumaCult‐Ex Plus media. For air–liquid interface (ALI) cultures, transparent inserts for a 24-well plate were used with a 0.4 μm pore size polyethylene terephthalate (PET) membrane and 0.3 cm^2^ culture area (Sarstedt). Inserts were coated with 1:100 Matrigel Growth Factor Reduced (GFR) Basement Membrane Matrix (Corning) dilution. Once confluent, submerged OM cells were passaged and seeded on coated 24-well transwell inserts at a seeding density of 4 × 10^4^–5 × 10^4^ cells and cultured in PneumaCult‐Ex Plus media for 2–4 days with media in both apical and basal chambers. Cells were monitored for confluence, and media was changed with fresh expansion media if required. Once a monolayer was observed, the cells were subjected to airlift by removing culture media from the apical side/compartment and at the same time replacing media in basal chamber to PneumaCult ALI medium (Stemcell Technology). The PneumaCult ALI medium was prepared according to the manufacturer’s instructions and supplemented with final concentrations of 4 µg/mL heparin (Paranova), 0.48 µg/mL hydrocortisone (Acros Chemicals), and 1% penicillin–streptomycin (Gibco). Cells were maintained in ALI for an additional 21 days for differentiation of primary cells to the pseudostratified epithelium. The ALI differentiation medium in the basal chamber was changed every 2–3 days and the apical chamber with the cells were washed with HBSS every 7 days to remove mucus secreted by the cells. These cells are called olfactory mucosa cell at air–liquid interface (OM-ALI).

### SARS-CoV2-propagation and purification

SARS-CoV-2 viral strains; Wuhan strain isolate wild type (WT, strain B.1), delta variant (strain B.1.617.2), omicron variant (B.1.1.529). SARS-CoV-2 was obtained under the Helsinki University Hospital laboratory research permit 30 HUS/32/2018 § 16. All viral work experiments were performed in BSL3 facilities at the University of Helsinki. The original stocks were propagated in VeroE6-TMPRSS2 cells (WT, delta and omicron). Detailed protocol for viral propagation has been previously described [18]. Viral stocks were stored at − 80 °C in Dulbecco’s modified Eagle’s medium, 2% fetal calf serum (FCS), 2 mM l-glutamine, and 1 × penicillin–streptomycin. Virus titers were determined by plaque assay in VeroE6-TMPRSS2 cells. All viral stocks were sequenced by next-generation sequencing, the presence of the furin cleavage site in the genome was confirmed.

### TEER measurements

Transepithelial electrical resistance (TEER) was measured to access the epithelial barrier integrity of the OM cultures at ALI every 7th day till the 21st day. An epithelial volt/ohm meter (EVOM2) from World Precision Instruments (Sarasota) was used with STX2 chopsticks electrodes for the TEER measurements, as described in [46]. TEER readings were obtained in triplicates for each line in OM-ALIs. TEER values were calculated as TEER (Ω/cm^2^) = (resistance total (Ω) – resistance blank (Ω)) × transwell insert surface in cm^2^.

### SARS-CoV-2 infection of the OM-ALI cultures

After 21 days, OM-ALIs in transwell inserts were transported to BSL3 facilities at the University of Helsinki for virus infections. Prior to infections, fresh media was changed to the basolateral compartments. ALI cultures from healthy donors or Alzheimer’s disease individuals were infected in triplicates. Briefly, OM-ALIs were inoculated with 50 μL of SARS-CoV-2 with either WT SARS-CoV-2 (1 × 10^5^ Plaque forming units/insert (PFU’s/insert)), delta variant (1 × 10^5^ PFU’s/insert), or omicron variant (1 × 10^5^ PFU’s/insert). Medium control (mock) was used as a negative control for infection. In addition, inhibition of the WT-SARS-CoV-2 viral cell entry was also tested by treating the cells with TMPRSS2 inhibitor Nafamostat (25 µM). The virus was applied to the apical surface of the OM-ALI to mimic viral infection in vivo. The basal chamber was not infected and contained only fresh PneumaCult ALI media. The infected OM-ALI cultures were incubated at 37 °C and 5% CO_2_ for 1 h, followed by aspiration of the virus and washing of the cells with Dulbecco’s phosphate buffered saline (D-PBS) three times. The last wash was saved and used for PCR which was carried out for quantification of SARS-CoV-2 viral RNA copies in the media. The infected OM-ALI cultures were then further incubated at 37 °C and 5% CO_2_ for 48 or 72 h. D-PBS apical washes were collected at 1, 24, 48, 72 h post-infection (hpi) to measure the viral RNA release from infected cells at later time points. Samples were stored at − 80 °C. Infected cells from OM-ALI cultures were harvested 48 hpi for messenger RNA (mRNA) sequencing or fixed at 72 hpi for immunofluorescence staining depending on the downstream assay.

### Quantification of viral RNA after infection

Infected cells from the apical side in transwell inserts were washed with PBS for 10 min. Apical PBS washes obtained from SARS-CoV-2 infected and mock-infected OM-ALI cultures at 1, 48, and 72 hpi were used to extract RNA using QIAamp Viral RNA Minikit (Qiagen) using the manufacturer’s protocol. Extracted RNA samples were used to perform SARS-CoV-2 quantitative RT-PCR using primers, a probe, and an in vitro synthesized control for RNA-dependent RNA polymerase (RdRp) as described earlier [47, 48]. SARS-CoV-2 RNA copies were accessed at each time point and a relative increase in viral load was determined.

### RNA preparation and RNA sequencing

Upon the completion of the SARS-CoV-2 infection, the basal culture media was removed, and the apical pseudostratified epithelium was washed three times in sterile D-PBS to remove any mucus or traces of leaked media. The OM-ALI cells were then collected in a lysis buffer of the AllPrep DNA/RNA/miRNA Universal Kit (Qiagen). Cells were pooled from three inserts to ensure the harvesting of enough total RNA for the sequencing. The samples were stored in a lysis buffer at − 80 °C prior to RNA extraction. Total RNA was extracted according to the manufacturer’s instructions. The Agilent 2100 Bioanalyzer and RNA 6000 Pico Kit were used to evaluate the integrity of the isolated RNA. RNA concentrations were measured with the Qubit fluorometer using the Qubit RNA HS Assay Kit (Invitrogen). 300 ng of total RNA of each sample was used for sequencing library preparation. Prior to the library preparation, ribosomal RNA was depleted with the QIAseq FastSelect RNA Removal Kit (Qiagen) according to the manufacturer’s instructions. RNA libraries were prepared using the QIAseq Stranded Total RNA Library Kit (Qiagen) according to the manufacturer’s instructions. Amplified libraries were subjected to quality control assessment on the Agilent 2100 Bioanalyzer using the High Sensitivity DNA Kit (Agilent). Concentrations of the libraries were measured with the Qubit fluorometer and the dsDNA HS assay kit (Invitrogen). Libraries were pooled at the equimolar concentration of 4 nM. Sequencing was performed on the Illumina NovaSeq 6000 platform using the S1 Reagent Kit (200 cycles). The 2 × 100 bp paired-end sequencing resulted in ~ 50 million reads per sample.

### RNA sequencing data processing and analyses

Sequencing data were adaptor trimmed using Trimmomatic [49] and aligned to human genome reference version hg38 using RNA-seq aligner STAR v2.7.10a [50]. Alternative contigs were excluded from the reference sequence. Specific parameters of STAR aligner were following: –outFilterType Normal –outFilterMultimapNmax 20 –alignSJoverhangMin 8 –alignSJDBoverhangMin 3 –outFilterMismatchNmax 999 –outFilterMismatchNoverReadLmax 0.2 –outFilterMismatchNoverLmax 0.05 –alignIntronMin 20 –alignIntronMax 1,000,000 –alignMatesGapMax 1,000,000 –outFilterIntronMotifs RemoveNoncanonicalUnannotated –twopassMode Basic. Reads were then assigned to RefSeq gene annotation using FeatureCounts v2.0.1 [51] with the following parameters: -largest Overlap -s 1 -p -B -P -d 30 -D 100000 -C -T 4. The number of reads belonging to genes was counted. From 52.8% to 64.8% of all reads were uniquely assigned to genes in each sample. This corresponds to at least 33.8 million reads per sample.

### Differential expression and pathway analysis

We performed the differential gene expression analysis between cells of the mock-treated AD and control libraries, mock-treated control and SARS-CoV-2 infected control cell libraries, mock-treated AD and SARS-CoV-2 infected AD cell libraries, and also between SARS-CoV-2 infected controls and SARs-CoV-2 infected AD cell libraries. For pathway enrichment analysis, we used the differentially expressed genes (DEGs) between control and AD with mock treatment and/or SARS-CoV-2 infection. PANTHER overrepresentation analysis for pathway enrichment was performed using PANTHER (version 17.0, released 2022-02-22, http://www.pantherdb.org/). Similarly, by using DEGs along with the fold changes we performed Ingenuity pathway analysis (IPA) for the identification of the altered canonical pathways. The IPA Analysis Match CL license used in this study was purchased from QIAGEN (https://www.qiagenbioinformatics.com/products).

### Immunocytochemistry and imaging

At 72 hpi with SARS-CoV-2, the OM-ALI cells were fixed using 4% paraformaldehyde (PFA). The PFA was added to both the apical and basal chambers for 10 min and then washed with D-PBS containing 0.2% bovine serum albumin (BSA) (Sigma). Fixed cells on the inserts were permeabilized with Triton X-100 (Sigma) at 1:100 dilution for 20 min and washed three times with the D-PBS + 0.2% BSA. Before primary antibody treatments, cells were blocked with 0.2% BSA in D-PBS for 30 min. Later, incubated overnight at 4 °C with predetermined concentrations of primary antibodies (Table 1) and subsequently washed three times with D-PBS to remove traces of non-binding primary antibodies. To visualize primary antibody binding, cells were treated with secondary antibodies for 3 h at room temperature (Table 1) and then washed with D-PBS + 0.2% BSA. For visualizing the nuclei, cells were stained with Hoechst (1:1000 dilution of 1 mg/mL stock) or bisbenzimide (1:1000 dilution of 1 mg/mL stock). After staining, the transwell membranes containing OM cells were removed from their inserts using a scalpel and peeled off from the bottom of the transwell using tweezers and mounted on glass slides using mounting media (Prolong Gold antifade reagent). The cells were facing upward and covered with 0.17-mm glass coverslips for imaging. Imaging was done using automated spinning disc CellVoyager™ CQ1 Benchtop High-Content Analysis System (Yokogawa) at the Imaging unit of the University of Helsinki at 10 and 20 × objectives, and Zeiss Axio Observer inverted microscope with LSM800 confocal module (Carl Zeiss AG) att the UEF Cell and Tissue Imaging Unit at 20, 40, and 63 × objectives. Image analysis was performed from 3D-confocal image stacks using the Cell Path Finder software inbuilt in the CQ1 microscope. Nuclei were automatically detected in the 3D stacks, and the fluorescence intensity of different epithelial cell markers analyzed within the nuclear volume expanded by 10 pixels in all directions. Classification of cells into positive and negative for a given marker was done with the same software using a manually determined threshold of fluorescence. Processing of the images was done using ZEN Blue version 3.2 (Zeiss) and open-source software ImageJ version 1.53q (Fiji).

**Table 1:** Key resources for immunostainings

Reagent or resource	Source	Identifier (catalogue number; lot number)
Primary antibodies		
Monoclonal anti-tubulin, acetylated antibody produced in mouse; Dilution: 1:2000	Sigma-Aldrich	T6793-100UL; 108923
Mouse monoclonal MUC5AC antibody (45M1); Dilution 1:200	Thermo Fisher Scientific	MA5-12178; WD3205962
Mouse monoclonal human Cytokeratin 18 antibody (810811); Dilution 1:50	R&D Systems	MAB7619; GR3268718-6
Goat anti-ACE-2 polyclonal antibody; Dilution 5 µg/mL	R&D Systems	AF933-SP; HOK0320051
Recombinant Rabbit monoclonal Anti-Neuropilin 1 antibody [EPR3113] Dilution 1: 250	Abcam	ab81321; 212288-45
ZO-1 Polyclonal Antibody; Dilution: 5 µg/mL	Invitrogen	40-2200; WA317222
SARS-CoV/SARS-CoV-2 Nucleocapsid Dilution: 1:2000	Kind gift by Jussi Hepojoki	Cantuti-Castelvetri et al., Science, 2020 [18]
Secondary antibodies		
Donkey anti-Goat Alexa Fluor-488 Dilution: 1:1000	Invitrogen	A-11055; 2,513,496
Donkey anti mouse Alexa Fluor555 Dilution: 1:500	Invitrogen	A-32773; VB302733
Goat anti rabbit Alexa Fluor-647 Dilution: 1:500	Invitrogen	A-31573; 2497486
Alexa Fluor-488 conjugated phalloidin	Invitrogen	A-12379; 1948083
Hoechst DNA stain	Thermo Fisher Scientific	62249; MF1423541

### Statistical methods and graphical illustrations

**Fig. 1:**
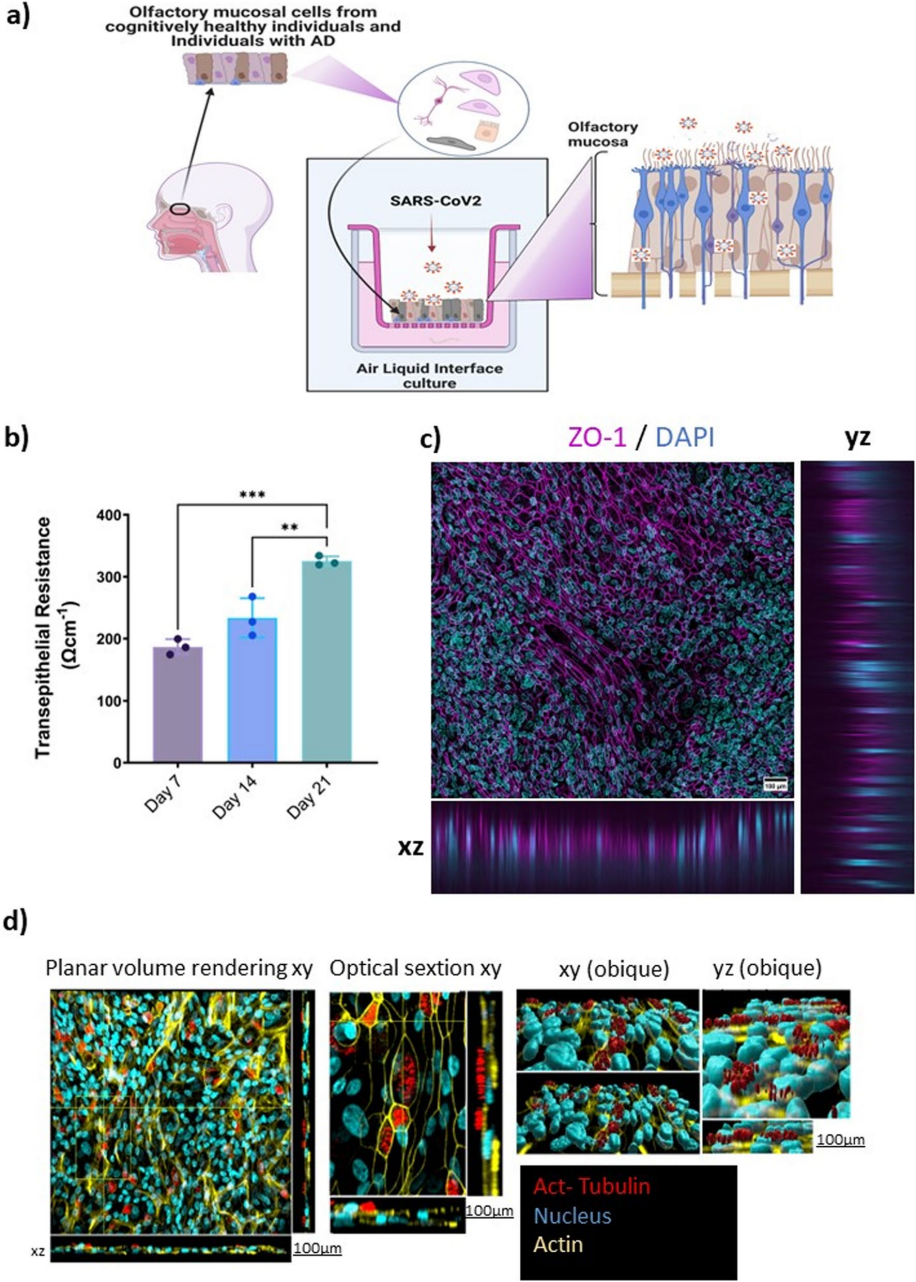
Characterization of the human primary olfactory mucosal cells at air–liquid interface. **a** Representation of the OM-ALI setup derived from human primary OM cells. **b** TEER measurement at days 7, 14, and 21 after initiation of OM-ALI cultures for cells derived from cognitively healthy control subjects. Graph shows mean with SD of *n* = 3 study subjects. Ordinary one-way ANOVA with Tukey’s multiple comparisons tests. **Indicates a *p*-value ≤ 0.005, *** indicates *p*-value ≤ 0.0005. **c**, **d** Representative immunostainings of OM-ALI cultures for the presence of zonula occludens-1 (ZO1), a tight junction marker; acetylated tubulin, a ciliary marker protein; and actin for the cytoskeleton of the cells. All imaged on 10 × objective; scale bar 100 µm

**Fig. 2:**
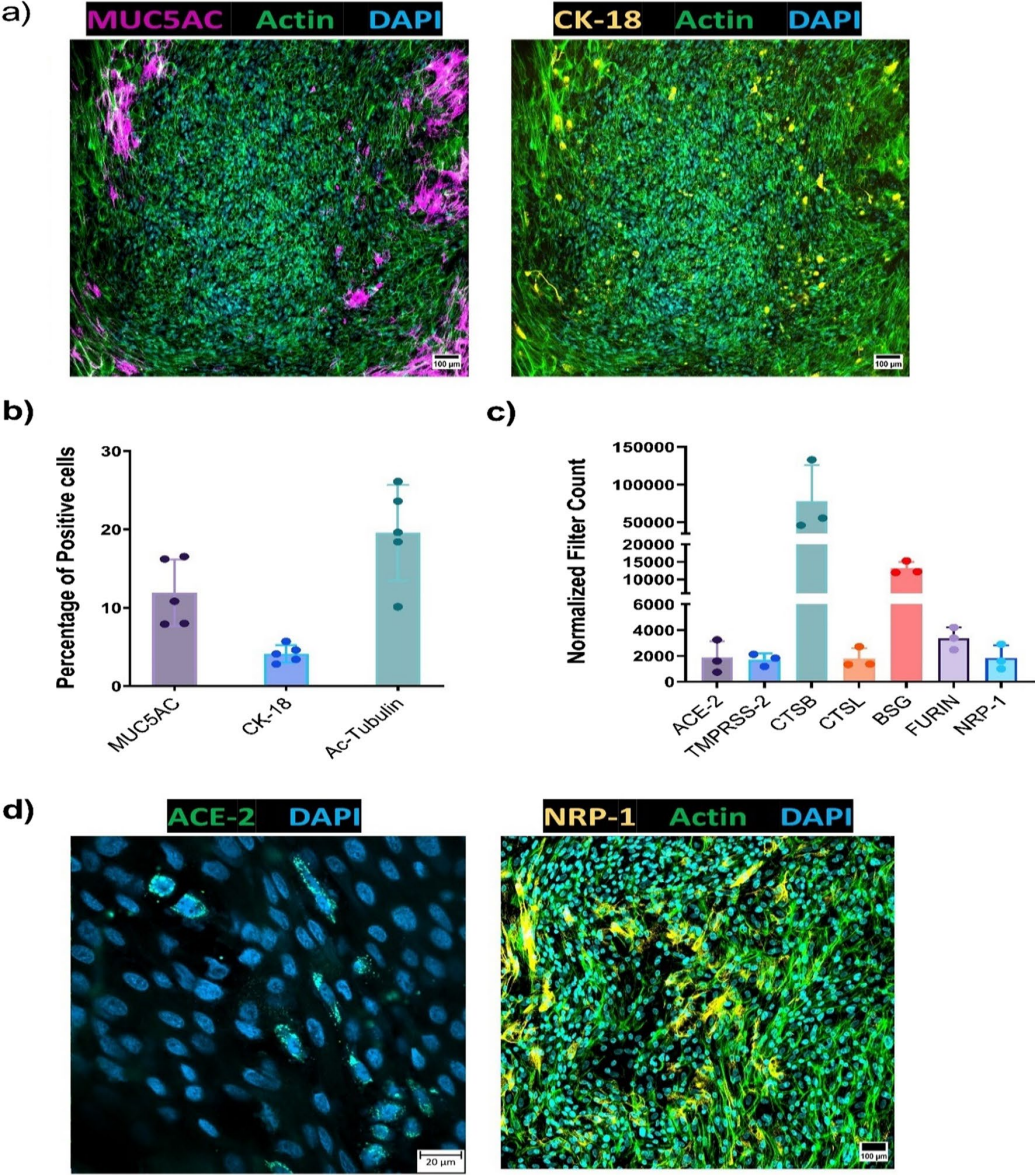
Human OM-ALI express non-neural epithelial cells and receptor proteins required for SARS-CoV-2 infection. **a** Representative immunostainings of OM-ALI cultures for the presence mucin 5AC (MUC5AC) for goblet cells; Cytokeratin 18 (CK-18) for sustentacular cells; and actin for the cytoskeleton of the cells. All imaged on 10 × objective; scale bar 100 µm. **b** Quantification of the immunostainings representing the percentages of positive cells for MUC5AC for goblet cells; Cytokeratin 18 (CK-18) for sustentacular cells; and Ac-tubulin (acetylated tubulin) for ciliated cells. Graph shows mean with SD of *n* = 3 for cognitively healthy controls (total of 5 images were analyzed, each dot represents percentage of positive cell in single image). **c** Normalized filter counts of genes involved in SARS-CoV-2 infection, obtained from bulk mRNA sequencing of mock-treated OM-ALI cultures derived from cognitively healthy controls. Graph shows mean with SD of *n* = 3. **d** Representative immunostaining images of ACE-2 and NRP-1 expression in the OM-ALIs. Imaged ACE-2 on 63 × (scale bar 20 µm), and NRP-1 on 10 × objective (scale bar 100 µm)

**Fig. 3:**
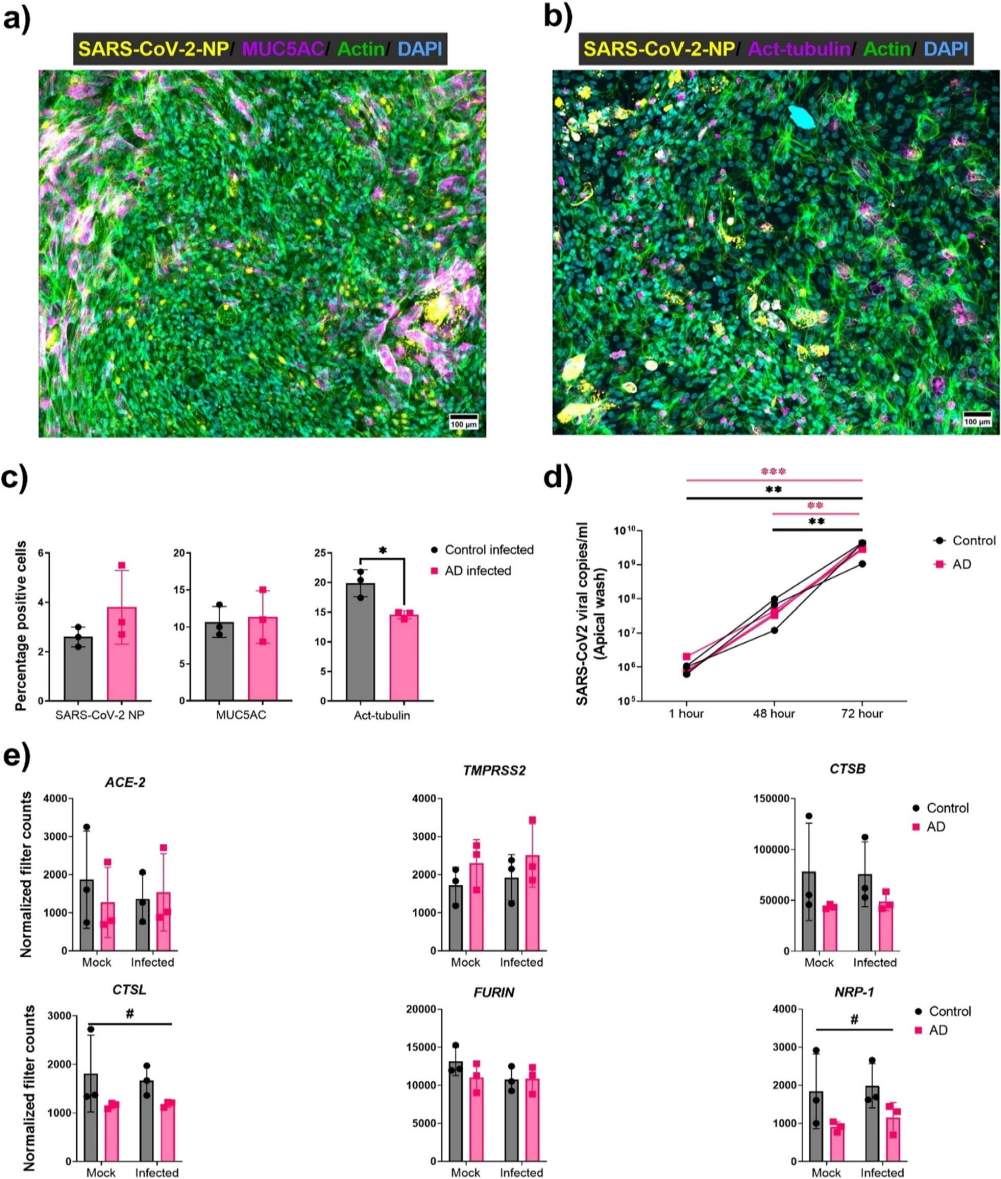
Human OM-ALI from AD and cognitively healthy individuals have similar susceptibility to SARS-CoV-2 infection. **a**, **b** Representative image of OM-ALIs at 72 hpi with SARS-CoV-2 (1 × 105 PFU). Immunostaining was done with SARS-CoV-2 NP (nucleocapsid protein); acetylated tubulin (ciliated cell marker); MUC5AC (goblet cell marker). Imaged on 10 × objective; scale bar 100 µm. **c** Quantification of the OM-ALIs at 72 hpi with SARS-CoV-2 (1 × 105 PFU) immunostainings representing the percentages of SARS-CoV-2 NP, Act-tubulin; MUC5AC in control and AD cells. Graph shows mean with SD of *n* = 3 for both control and AD cells. Unpaired two-tailed t-test. *Indicates *p*-values ≤ 0.05. **d** Quantification of SARS-CoV-2 RNA copies released from the infected control and AD OM-ALIs at 1, 48, and 72 hpi. Graph shows mean with SD of *n* = 3 donors for both control and AD OM-ALIs. **Indicates *p*-values ≤ 0.01, ***indicates *p*-values ≤ 0.001 by two-way ANOVA with Tukey multiple comparison. e) Normalized filtered counts of genes for key receptors and proteins involved in SARS-CoV-2 infection obtained from bulk mRNA sequencing of mock and infected OM-ALI cultures from control and AD. Graph shows mean with SD of *n* = 3 for both control and AD cells. Ordinary two-way ANOVA test; # indicates the disease effect and *p*-values ≤ 0.05

**Fig. 4:**
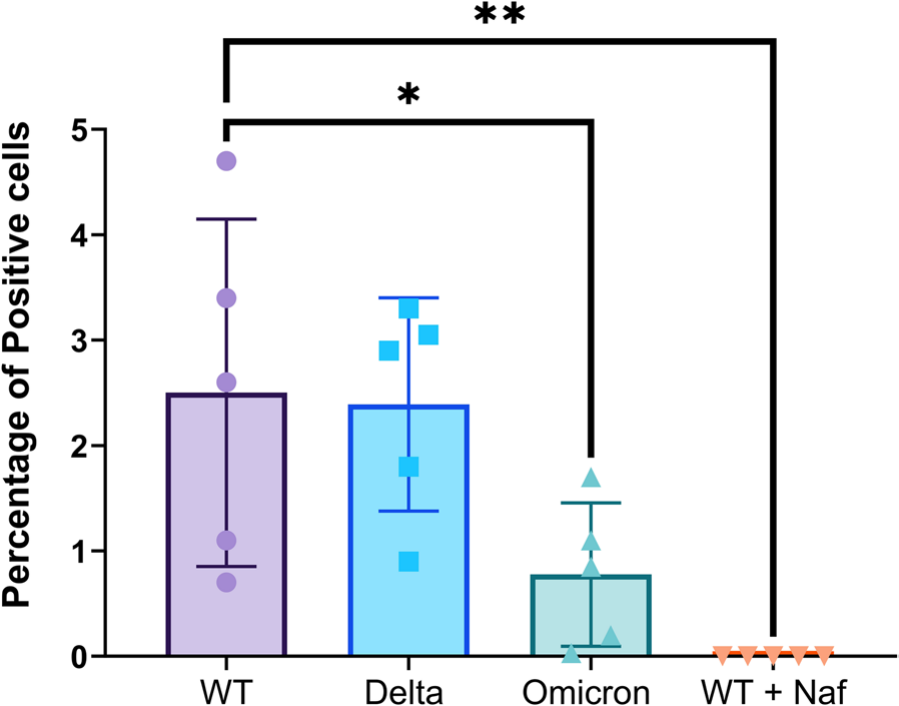
Human OM-ALI cells from healthy individuals exhibit variant-specific susceptibility to SARS-CoV-2 infection. Quantification of the of viral NP-positive OM-ALIs at 72 hpi with WT-SARS-CoV-2 (1 × 105 PFU), delta variant (1 × 105 PFU’s), omicron variant (1 × 105 PFU’s), and inhibition of WT infection with pre-treatment of Nafamostat (25 µM). Graph shows mean with SD of *n* = 3 for cognitively healthy controls (total of 5 images were analyzed, each dot represents percentage of positive cell in single image). One-way ANOVA with Dunnett’s multiple comparisons tests; *indicates *p*-values ≤ 0.05 and **indicates *p*-values ≤ 0.01

The GraphPad Prism 9.4.1 (GraphPad Software Inc.) software was used for the statistical analysis of the data. Statistical analysis methods used for different comparisons are indicated in figure legends. Error bars in Figs. 1, 2, 3 and 4 represent standard deviation (SD). Statistical significance was assumed for *p*-values ≤ 0.05. The graphical illustrations were created with BioRender.com.

## Results

### Primary human OM cells efficiently differentiate into pseudostratified epithelium in ALI cultures in vitro

To study SARS-CoV-2 infection in differentiated OM-ALIs, we first established ALI culture conditions for cells derived from OM biopsies (Fig. 1a). OM-ALIs were cultured under ALI conditions for 21 days and then characterized for epithelial barrier function and tight junctions. Trans-epithelial resistance (TEER) was measured to determine the polarization and integrity in cultures 7, 14, and 21 days after initiation of the ALI cultures. A significant increase in TEER values (*F* = 36.39, *p* = 0.0004) was observed at day 21 (mean 325.12 Ω/cm^2^, SD = 20.48) as compared to the day 7 TEER values (mean 186.56 Ω/cm^2^, SD = 33.21) (Fig. 1b). To characterize the ALI model further, the cells were immunostained for ZO-1 to confirm tight junctions, and for acetylated tubulin to detect the presence of cilia. (Fig. 1c, d, Additional file 1: Fig. S1a, b). Our results showed the presence of pseudostratified epithelium with tight junction to mimic a physical barrier at the OM.

### Primary human OM cells express non-neural epithelial cells in ALI cultures and express ACE-2 and other entry receptors required for SARS-CoV-2 infection

We further characterized the cell types expressed in the OM-ALI. Immunocytochemical staining of cell type-specific markers demonstrated that the ALI cultures contain populations of different cell types present in the human OM in vivo, including an average of 12% of goblet cells (Mucin 5AC + cells), and 4–5% sustentacular cells (Cytokeratin-18 + cells) (Fig. 2a, b; Additional file 1: Fig. S1c, d). Quantification of the immunostainings showed an average of 20% of cells within the differentiated OM-ALI cultures express apical ciliary markers (Fig. 2b). We further characterized differentiated OM-ALI cultures for expression of entry receptors required for SARS-CoV-2 infection. Global mRNA sequencing data analysis from uninfected cells indicated the presence of the *ACE-2, NRP-1, TMPRSS2,* Cathepsin B (*CTSB*), Cathepsin *L (CTSL*), basigin (*BSG*)*,* and furin mRNAs (Fig. 2c), all of which are implicated to be important for SARS-CoV-2 infection [52–56]. SARS-CoV-2 is known to primarily target ACE-2; cellular transmembrane receptors for binding to the host cell membrane [52], whereas NRP-1 has also been shown to enhance infection of SARS-CoV-2 in ACE2-expressing cells [18]. Therefore, immunocytochemical staining of differentiated OM-ALI cultures for the ACE-2 receptor and NRP-1 proteins was performed to validate the expression the of most important entry receptors required for SARS-CoV-2 infection (Fig. 2d).

### SARS-CoV2 infects OM cells of both cognitively healthy donors and individuals with AD

Having characterized the ALI cultures and knowing they express entry receptors required for SARS-CoV-2 infection, we next sought to determine the infectability of the cells and to compare how cells from individuals with AD may differ from cells derived from cognitively healthy individuals. After growing cells in ALI for three weeks, the AD and cognitively healthy control cells were subjected to apical infection with the SARS-CoV-2-WT (1 × 10^5^ PFU) for 72 h. The representative image of infections with SARS-CoV-2 nucleocapsid protein (NP) co-stained with MUC5AC (Fig. 3a, Additional file1: Fig. S1f), and acetylated tubulin (Fig. 3b), indicating the presence of the virus in the OM-ALIs. Furthermore, co-staining with acetylated tubulin indicated the presence of viral NP in the apical epithelium (Fig. 3b, Additional file 1: Fig. S1e).

Viral infections in OM-ALI cells showed no significant differences in viral NP or MUC5AC (goblet cell marker) positive cells between the groups (Fig. 3c). However, infected OM-ALI cells show a significant reduction in the acetylated tubulin (ciliary marker) in AD individuals-derived OM-ALI cells (*t* = 3915, *df* = 4, *p* = 0.0173 (Fig. 3c).

In addition to quantifying the numbers of SARS-CoV-2 positive cells, we also monitored secreted viral RNA copies released from the apical side of the OM-ALI cultures at 1, 48, and 72 hpi. In both healthy control and AD OM-ALI cultures the viral RNA levels increased after SARS-CoV-2 infection in a time-dependent manner, confirming the viral replication and propagation with the infected cells. However, no significant differences were observed between viral RNA levels released from the apical side of the OM-ALI cultures at any observed time point between cells derived from cognitively healthy controls and individuals with AD (Fig. 3d). We also determined the expression of SARS-CoV-2-associated genes in infected and mock-treated cell samples. No significant differences in mRNA expression *of ACE-2, TMPRSS2, CTSL, BSG, Furin,* and *NRP-1* were observed between the cells derived from cognitively healthy control subjects and AD individuals in mock-treated or SARS-CoV-2 infected samples (Fig. 3e). Interestingly, the two-way ANOVA test showed that the presence of disease (AD) had a significant effect on alteration in gene expression levels of *CTSL* (*F* = 5.406; *p* = 0.049) and *NRP-1* (*F* = 6.432; *p* = 0.034) (Fig. 3e).

Next, cells of the cognitively healthy donor were used to determine the susceptibility of the cultures to different SARS-CoV-2 variants. For this experiment, we infected the OM-ALI cells with WT, delta, and omicron variants of SARS-CoV-2 (Fig. 4). A recent review comparing published data on the contribution of genetic variants to the incidence of anosmia concluded that the omicron variant causes less olfactory dysfunction than the other variants, indicating that WT and delta have a broader invasive potential in the OE [57]. Coinciding with these data, our results also showed differences in the infectibility of OM-ALI with the investigated variants of SARS-CoV-2 with one-way ANOVA (*F* = 7.202, *p* = 0.0028). Furthermore, the OM-ALI cultures were less susceptible to infection with the omicron variant as compared to the WT SARS-CoV-2 when determined by the percentage of cells positive for the virus variants in question (*p* = 0.0439) (Fig. 4); whereas no significant difference was observed in the percentage of positive cells when comparing the WT and delta variants. Lastly, inhibition of the SARS-CoV-2 viral infection with short pre-treatment of cells with TMPRSS2 inhibitor Nafamostat resulted in complete inhibition of infection by the WT SARS-CoV-2 (*p* = 0.0038) (Fig. 4). This suggests that in the OM-ALI viral entry into the cell is dependent on proteolytic cleavage of the spike protein with TMPRSS2 after the virus binds to the ACE-2 receptor [58].

### Distinct gene expression changes in OM-ALI cells from Alzheimer’s individuals infected with SARS-CoV-2

**Fig. 5:**
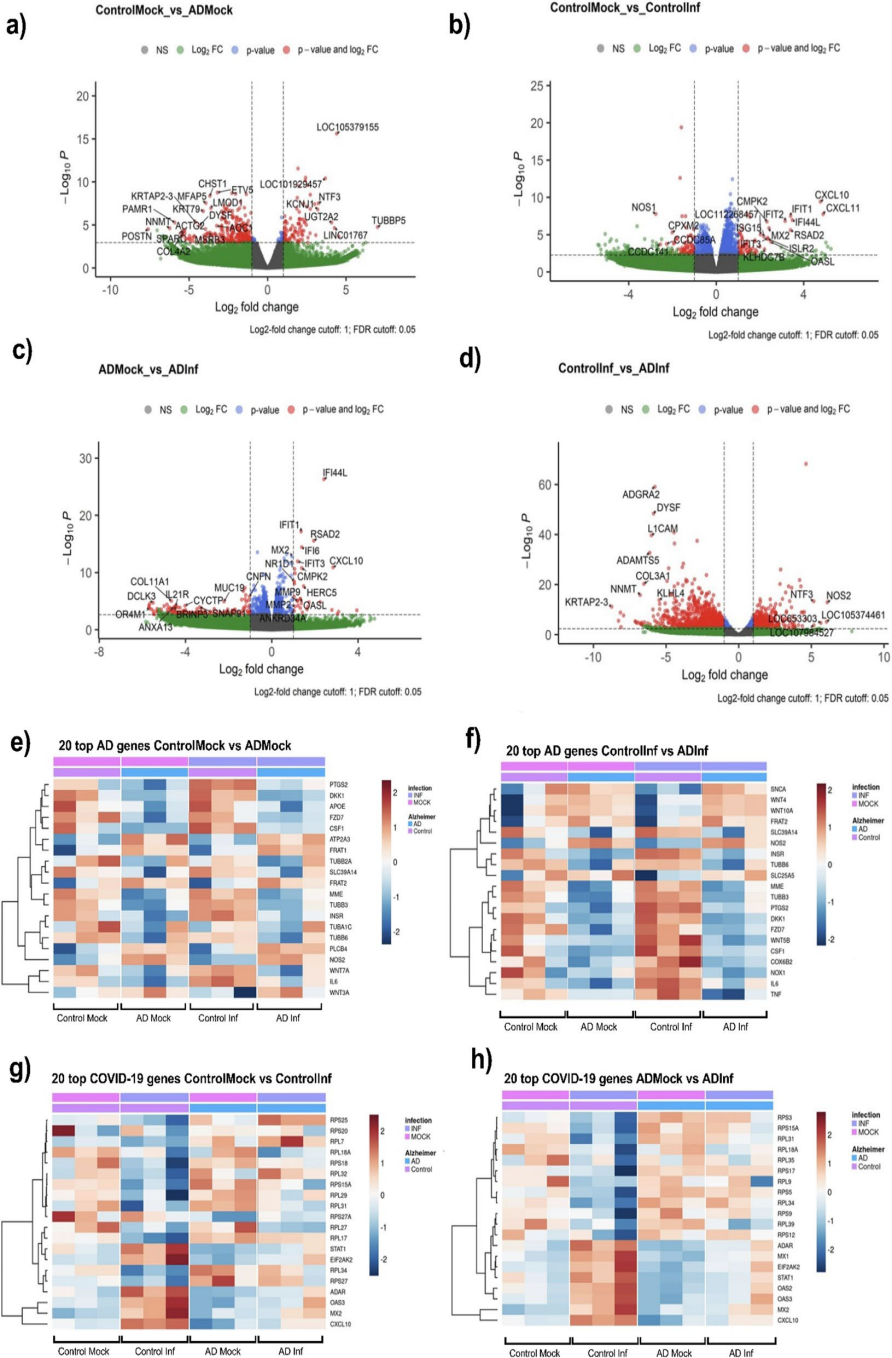
AD alters OM-ALI cell response to SARS-CoV-2 via transcriptomic changes. **a**–**d** Volcano plots showing differentially expressed genes (DEGs) between **a** control mock and AD mock, genes with Log2 fc of ≤ -3 or ≥ 3 and FDR ≤ 0.01 are highlighted; **b** control mock and control infected, genes with Log2 fc of ≤ -2 or ≥ 2 and FDR ≤ 0.01 are highlighted; **c** AD mock and AD infected, genes with Log2 fc of ≤ − 2 or ≥ 2 and FDR ≤ 0.01 are highlighted; **d** control infected and AD infected, genes with Log2 fc of ≤ − 5 or ≥ 5 and FDR ≤ 0.01 are highlighted. The red dot indicates Log2 fc cutoff 1 and FDR cutoff 0.05. **e**, **f** Heatmaps showing Alzheimer’s disease KEGG pathway (hsa05010) associated top 20 DEGs in; **e** control mock and AD mock; **f** control infected vs AD infected. **g**, **h** Heatmaps showing KEGG human coronavirus disease pathway (hsa05171) associated DEGs in **g** control mock and control infected and in **h** AD mock and AD infected. *fc* fold change, *FDR* false discovery rate

Having established an in vitro representative model for human OM cells that are susceptible to SARS-CoV-2 infection, we sought to elucidate the transcriptomic alterations caused by SARS-CoV-2 infection in cognitively healthy individuals and individuals with AD. For this, we performed bulk mRNA sequencing on OM cells that were either infected with WT SARS-CoV-2 (1 × 10^5^ PFU’s) for 48 h or received mock treatment. Principal component analysis and differential gene expression analysis of the sequencing results revealed profound differences between control and AD cells. We discovered a total of 427 (138 upregulated and 289 downregulated) significantly differentially expressed genes (DEGs) (FDR < 0.05) in mock-treated AD OM-ALI cells when compared to mock-treated control OM-ALI cells (Additional file 5: Table S1). Figure 5a depicts the DEGs representing the baseline differences between mock-treated controls and AD individuals. Based on the log2-fold change, the five most upregulated genes in AD individuals are *TUBBP5* (Tubulin beta pseudogene 5), *NOS2* (Nitric Oxide Synthase 2), *UGT2A2* (UDP Glucuronosyltransferase Family 2 Member A2), *NTF3* (Neurotrophin 3), *and KCNJ1* (Potassium Voltage-Gated Channel Subfamily J Member 1), while *POSTN* (Periostin), *FN1* (Fibronectin 1), *NNMT* (Nicotinamide N-Methyltransferase), *PAMR1* (Peptidase Domain Containing Associated with Muscle Regeneration 1), *and COL4A1* (Collagen Type IV Alpha 1 Chain) are the five most downregulated genes. Interestingly, *UGT2A1* and *UGT2A2* have recently been implicated in COVID-19-associated loss of smell and taste [59].

Next, we assessed the impact of SARS-CoV-2 infection on the gene expression of control and AD OM-ALI cells. Infection of cells derived from cognitively healthy controls with SARS-CoV-2 resulted in 1797 DEGs (FDR < 0.05), out of which 1113 were upregulated and 684 were downregulated in comparison to mock-treated cells (Fig. 5b, Additional file 5: Table S2). In cells derived from individuals with AD, SARS-CoV-2 infection resulted in 1176 DEGs, out of which 624 were upregulated and 552 downregulated (Fig. 5c, Additional file 5: Table S3). Interestingly, our analysis showed 1971 (790 upregulated 790 and 1181 downregulated) differentially expressed genes in infected AD OM-ALI when compared to infected control OM-ALI cells (Fig. 5d, Additional file 5: Table S4). Many DEGs suggest that underlying AD pathology altered the responses of cells to SARS-CoV-2 infection.

As expected, SARS-CoV-2 infection in control OM-ALI cells showed that the highest fold changes were observed in genes that are involved in the antiviral immune response*: CXCL11* (C–X–C motif chemokine ligand 11, fc = 4.8), *CXCL10* (C–X–C motif chemokine ligand 10, fc = 4.7), and *IL6* (interleukin 6*,* fc = 1.76). Moreover, in the antiviral signaling response, *IFI44* (Interferon Induced Protein 44, fc = 3.47), *IFIT1* (Interferon Induced Protein with Tetratricopeptide Repeats 1*,* fc = 3.4)*, RSAD2* (Radical S-Adenosyl Methionine Domain Containing 2, fc = 3.3), *IFIT2 (*Interferon Induced Protein with Tetratricopeptide Repeats 2, fc = 3.15), *OASL* (2'-5'-Oligoadenylate Synthetase Like, fc = 2.4), *MX2* (MX Dynamin Like GTPase 2*,* fc = 2.4), ISLR2 (Immunoglobulin Superfamily Containing Leucine Rich Repeat Protein 2, fc = 2.3), and *IFIT3* (Interferon Induced Protein with Tetratricopeptide Repeats 3, fc = 2.3) exhibited increased expression. Both antiviral immune response and interferon-mediated signaling are characteristic features of SARS-CoV-2 infection reported in the literature in vitro, ex vivo, and in vivo [60–62]. However, in AD OM cells the upregulation of genes associated with the immune response and interferon-mediated signaling after infection with WT-SARS-CoV-2 were less drastic, and only a few genes were observed in the top 20 significant DEG when compared to mock-treated AD OM-ALI cells, including *CXCL10* (fc = 2,8), *IFI44L* (fc = 2,4), *RSAD2* (fc = 1,9), *IFI6* (fc = 1,3), *IFIT1* (fc = 1,3), and *OASL* (fc = 1,32). Surprisingly, 4 out of 10 most upregulated DEGs, and 8 out of 10 most downregulated DEGs in AD-infected OM-ALI were non-coding RNAs (ncRNA) which was not seen in the control-infected OM. Among the most significant differentially expressed ncRNAs (*NEAT1, TALAM1, GAS5*) have been previously reported to be associated with SARS-CoV-2 infection [63–65].

To investigate the effect of AD pathology in the mock and infected samples, we compared numbers of RNA sequencing reads from mock control and AD cells, and infected controls and AD samples to the Kyoto Encyclopedia of Genes and Genomes (KEGG) database of genes involved in Alzheimer’s disease pathway (hsa05010). Figure 5e shows the top 20 DEGs in mock cells from cognitively healthy and AD individuals from the KEGG AD pathway. Similarly, we also identified top DEGs in infected cells from cognitively healthy and AD individuals from the KEGG AD pathway (Fig. 5f). Furthermore, to investigate the SARS-CoV-2 infection effect, we showed top differential transcriptomic signatures of SARS-CoV-2 infection compared to KEGG human Coronavirus disease pathway (hsa05171) in cognitively healthy and AD individuals (Fig. 5g, h). Interestingly, the top AD pathway-associated DEGs between the control mock and AD mock are *PTGS2* (Prostaglandin-Endoperoxide Synthase 2), *DKK* (Dickkopf-related protein), and *APOE* (Apolipoprotein E), whereas AD-pathway-associated DEGs in control infected and AD infected include *SNCA* (α-synuclein), *WNT* (Wingless-related integration) site 4 and *WNT10A*. On the other hand, the top SARS-CoV-2 pathway-associated DEGs between the control mock and control infected are *RPS* (ribosomal protein) *S25, RPS20*, and *RPL7*, whereas SARS-CoV-2-pathway-associated DEGs in AD mock and AD-infected cells include *RPS3, RPS15A*, and *RPL31.*

We further performed PANTHER pathways overrepresentation analysis on DEGs between infected and non-infected control and AD cells. Interestingly, we found only the integrin signaling pathway and Alzheimer’s disease-presenilin pathway that were significantly enriched. In addition, analysis of all the significant DEGs demonstrated other pathways implicated in AD (Additional file 2: Fig. S2A). This indicates the utility of patient-derived cells in understanding SARS-CoV-2 infection. Interestingly, SARS-CoV-2 infection in control and AD cells enriched similar pathways associated with SARS-CoV-2. However, in the SARS-CoV-2-infected AD cells, more AD-associated pathways were enriched (Additional file 2: Fig. S2B). Enriched pathways include Inflammation mediated by chemokine and cytokine signaling pathways, cadherin signaling pathways, and Wnt-signaling pathways. Furthermore, the numbers of DEGs associated with the common AD pathways were significantly increased in SARS-CoV-2 infected AD cells than in the mock-treated AD (Additional file 2: Fig. S2C).

**Fig. 6:**
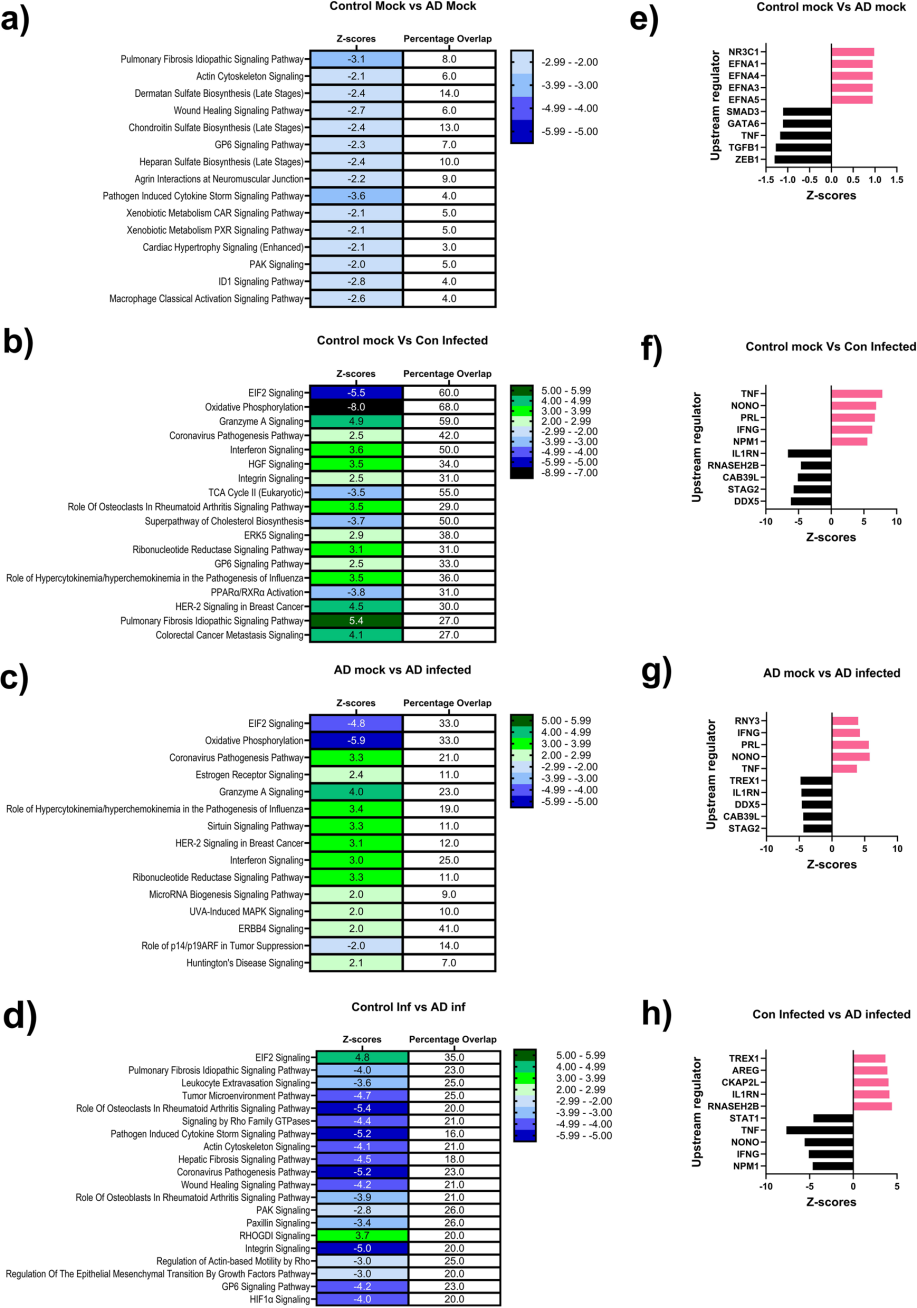
AD OM-ALI cells show distinct biological changes post-SARS-CoV-2 infection. Top signaling pathways in canonical pathway comparison between all exposure groups with the percentages overlap of pathway genes with DEGs: **a** mock-treated controls and SARS-CoV-2 infected controls; **b** mock-treated AD and SARS-CoV-2 infected AD; **c** mock-treated control and mock-treated AD; **d** SARS-CoV-2 infected control and SARS-CoV-2 infected AD cells. The rankings were based on Fisher's exact test and pathways are presented with the highest significance on the top and displayed along in decreasing order of significance from the top. The cutoff for statistical significance was a *p*-value ≤ 0.05 and a biological significance indicated by *Z*-score ≤ − 2 or ≥ 2. *n* = 3 control and *n* = 3 AD OM-ALI cultures for all data. **e**–**h** Indicates upstream regulators associated to DEG in the **e** control mock and AD mock; **f** control mock and control infected; **g** AD mock and AD infected; h control infected and AD infected. *Y*-axis indicates the upstream regulator network, and the x-axis represents the activation *Z-*score

Core analyses in IPA were performed for all the significant DEGs in each data set to identify pathways associated with differential gene expression patterns. Associated networks determined by the analysis were used to identify upstream regulators. Altered pathways that were associated with the DEG in between the mock-treated control and AD OM-ALI cells were related to an extracellular matrix organization, proteoglycans synthesis dysregulation (associated with Aβ deposition), synaptic dysfunction, wound healing, and inflammation (Fig. 6a, Additional file 5: Table S5). Furthermore, we found AD-associated upstream regulators including *ZEB1* (Zinc Finger E-Box Binding Homeobox), *TGFB1* (Transforming Growth Factor Beta 1), and *TNF* (Tumor Necrosis Factor) that are inhibited and *NR3C1* (Nuclear Receptor Subfamily 3 Group C Member 1), and various *EFNA* (ephrin A’s) activated in mock AD cells. All these upstream regulators are linked to AD. The figure shows the top five statistically significant inhibited and activated upstream regulators in the mock-treated control and AD OM-ALI cells (Fig. 6e, Additional file 5: Table S6).

In both control and AD cells, as expected, infection with SARS-COV-2 was linked to the Coronavirus Pathogenesis Pathway or the Coronavirus Replication Pathway. In IPA, core analysis for comparison of SARS-CoV-2 infection with the representative mock treatment in control and AD ranked eIF2 signaling as the most affected pathway (Fig. 6b, c). Viruses hijack the host cell machinery to complete viral replication and protein synthesis. In response, host cells turn off these systems, which is thought to be an integrated stress response. The stress response induces translational silencing via phosphorylation of eIF2 (eukaryotic initiation factor-2) [66]. Therefore, sustained phosphorylation of eIF2 inhibits host or viral protein synthesis. Interestingly, a recent study also showed downregulation of the elF2 signaling pathway in the transcriptomic analysis of nasopharyngeal swabs derived from COVID-19-infected patients [67]. Furthermore, IPA analysis showed SARS-CoV-2 infection in control and AD cells to be associated with mitochondrial dysfunction and downregulation of the oxidative phosphorylation pathway (Additional file 5: Tables S7, S8). In both infection groups, a similar trend of alterations in the top three pathways was observed. However, overall coverage of the pathway with the DEGs in control OM-ALI cells was almost double that of the AD OM-ALI cells after infection. Like the canonical pathways, upstream regulators between SARS-CoV-2 infected control and AD OM-ALI cells are the same, indicating a similar pattern of infection (Additional file 5: Tables S9, S10). The upstream regulators are mainly involved in antiviral immune response and inflammation. The top 10 biologically and statistically significant upstream regulators between control mock and control infected (Fig. 6f) and AD mock and AD infected (Fig. 6g) are shown.

Furthermore, core IPA analysis of the DEGs between control and AD cells post-infection confirms significant biological differences between the up- or downregulation of pathways (Fig. 6d). For example, elF2 signaling was downregulated in both control and AD cells upon infection. However, the IPA of DEGs comparing control infection with the AD infection showed less effective downregulation of the elF2 pathway in the AD OM-ALI cells as compared to control OM-ALI. Interestingly, pathways related to an extracellular matrix organization, wound healing, integrin signaling, and inflammation which were already downregulated in the mock-treated AD cells were further downregulated after infection (Fig. 6d, Additional file 5: Table S11). Although as shown above, the top upstream regulators were common for infection of the control and AD cells, both quantitative and biological activity differences of upstream regulators between SARS-CoV-2 infected control and AD OM-ALI were observed (Fig. 6h, Additional file 5: Table S12).

## Discussion

The OM, situated at the rooftop of the nasal cavity, is in direct contact to inhaled air and the particles present in it. Previous studies have shown infection of the OM with the SARS-CoV-2, and this has been investigated in ex vivo human olfactory biopsies from infected individuals [68, 69]. However, analyses of viral replication and pathophysiological processes caused by infection of the OM cells are limited. Furthermore, there is a lack of understanding of viral infection processes and possible differential responses of the infected cells to SARS-CoV-2 in underlying neurological disease conditions, i.e., AD. According to the current information, this paper presents the first efforts to address these knowledge gaps and to develop a physiologically relevant human-derived 3D in vitro model of the OM. We present evidence that human OM biopsies-derived cell cultures, when grown in ALI, recapitulate key features of the OM in vivo, and can be used to model viral infections under controlled experimental conditions. Furthermore, in this study, we present new insight into the infectability of the OM cells derived from AD individuals in comparison to those of cognitively healthy individuals and decipher the transcriptomic crosstalk between AD and SARS-CoV-2 infection at the OM.

In this study, we established a novel 3D ALI culture of the human OM. Characterization of cells derived from OM biopsies taken from cognitively healthy individuals and those affected by AD after three weeks in ALI indicated the presence of pseudostratified epithelium and cells expressing cilia. Furthermore, our results showed that the OM cells grown in ALI formed a barrier, confirmed through TEER measurement and expression of tight junction markers. Although we were unable to identify neither immature nor mature olfactory neurons, we confirm that sustentacular cells, basal cells, and mucous-producing cells are among the different cell types expressed in the OM-ALI cultures.

Our previous evidence from single-cell transcriptomic analysis of traditional 2D cultures of OM cells revealed the presence of AD-associated pathology in the OM cells derived from individuals with AD [30]. Consistent with that, in this study, the transcriptomic profile of the AD OM-ALI cells is also distinct from that of cognitively healthy control cells. We report a total of 427 DEGs between the control mock and AD mock OM-ALI cultures and further shortlist the top 20 DEGs that are associated with Alzheimer’s disease KEGG pathway (hsa05010). Interestingly, several of these top DEGs were found to be commonly attributed to AD in other cells or in vivo, i.e., *DKK1* [70], FZD7 (Frizzled-7) [71], *PTGS2* [72], and *APOE* [73]. Furthermore, enrichment of AD-associated pathways, i.e., integrin signaling pathway, Alzheimer’s disease-presenilin pathway, and Wnt-signaling pathway, were observed in pathway analysis of DEGs between non-infected control and AD OM-ALI cells. In addition, key upstream regulators that are linked to the DEGs found in non-infected AD cells correspond to the key phenomena in AD including inflammation, oxidative stress, regulation of Aβ deposition, neuronal dysfunction, and synaptic plasticity. Therefore, we believe that the OM-ALI culture of cells of individuals with AD exhibits pathological features associated with the disease.

Importantly, the OM-ALI cultures express ACE-2, the main entry receptor of SARS-CoV-2—the expression of this receptor has also been previously demonstrated ex vivo in the human OE [10, 13, 74]. Aside from the role of ACE-2 in facilitating viral entry, some research has put forth the Neuropilin-1 receptor (NRP-1) as an alternative means of entry for SARS-CoV-2 [17]. The expression of NRP-1 in the OE is not limited to certain cell type and as is the case with ACE-2 [18]. In coherence with the evidence ex vivo, in this study, we confirmed that OM-ALI cells also express the NRP-1 receptor. Furthermore, OM-ALI cells expressed genes for all the characteristic proteins that are important for viral entry and infection, such as TMPRSS-2, CTSB, CTSL, NRP-1, BSG, and furin.

Evidence to date indicates that SARS-CoV-2 infects the non-neural cells of the OE, mainly the sustentacular cells surrounding the olfactory sensory neurons (OSNs) [68, 75]. Sustentacular cells provide functional and structural support to the OSNs and hence play a crucial role in olfaction [76, 77]. Infection of sustentacular cells leads to detrimental effects on the OE, which may lead to olfactory function impairment. This can happen either through a direct impact on the uniformity of the OE or indirectly through affecting the metabolic and functional activity of the OSNs. Research conducted on Syrian hamsters by Bryche et al*.* showed that the loss of sustentacular cells caused by the virus resulted in the desquamation of the OE, the recruitment of immune cells, and the loss of OSN cilia [78]. Another study by Zazhytska et al*.* reported significant damage to the OE, although there was little infection of the OSNs [69]. The study also documented the downregulation of sustentacular cell-specific markers, followed by the downregulation of OSN-specific genes and related signaling pathways that play a role in the sense of smell. Consistent with the others, our study demonstrates infection of apical cells of the OM-ALI, including ciliary cells and non-neuronal epithelial cells.

In addition to the above-mentioned effects, previous research has corroborated the involvement of immune cells in the infection of OM. Infection of sustentacular cells located in the OE prompts the production of pro-inflammatory cytokines as a defensive reaction against viral invasion. However, these pro-inflammatory cytokines could provoke harm to the OE, and thereby induce dysfunction of the OSNs. In this study, acute infection in the OM-ALI cells derived from cognitively healthy controls and AD individuals led to a robust immune response and upregulation of the pro-inflammatory response. It has been observed in patients with long COVID that even after the resolution of SARS-CoV-2 infection in the OM, gene expression changes remain in the sustentacular cells. These changes suggest a reaction to the ongoing inflammation signaling and are accompanied by a reduction in the number of OSNs [79]. Since our ALI culture model does not include neurons, it is possible that in vivo the OSNs can also be infected, although this may be limited, as earlier reported [16, 80]. It is herein not possible to completely understand the crosstalk between sustentacular cells and OSNs, however, our transcriptomic data from infected OM-ALI cells indicate downregulation of several genes that are linked to neuronal plasticity, axonal guidance, and neuronal survival, thereby supporting the hypothesis that infection of the supporting cells of the OM can induce secondary harmful effects on OSN functions.

The current study presents the transcriptomic landscape of WT SARS-CoV-2 infection in human OM cells. As expected, differential gene expression analysis of SARS-CoV-2 infected OM-ALI cells revealed COVID-19 pathogenesis pathway activation along with alterations in genes involved in inflammation and antiviral immune response through interferon signaling. Pathway analysis of DEGs in SARS-CoV-2 infected OM-ALI cells also revealed alterations of genes involved in oxidative phosphorylation and mitochondrial function. Others have reported mitochondrial dysfunction in infected brain cells, which has been attributed to the neuropathogenesis of SARS-CoV-2 infection [81]. Furthermore, mitochondrial dysfunction is a hallmark of many neurodegenerative diseases, including AD, and alterations in mitochondrially located genes and mitochondrial function in OM cells of individuals with AD have been previously demonstrated [30]. It is plausible that increased mitochondrial stress and reduced oxidative phosphorylation resulting from SARS-CoV-2 infection in the OM further intensify oxidative stress and may exacerbate the pro-inflammatory response of the OM, which could potentially be damaging to the epithelial barrier.

A recent study suggested that modification of the SARS-CoV-2 spike protein potentially alters cell tropism and interaction with proteins that promote virus uptake [82]. That also corresponds with the prevalence data from the emerging variants of SARS-CoV-2 which indicate that there are differences in the incidence of anosmia in COVID-19-affected individuals with certain variants [57]. In this study, we demonstrated the changes in the infectibility of the OM-ALI cultures with the different variants of SARS-CoV-2. Interestingly, OM-ALI cells showed greater susceptibility to infection with SARS-CoV-2-WT as compared to the omicron variant of SARS-CoV-2, whereas the delta variant did not differ significantly from the infection with the WT virus. Reduction in the number of infected cells of OM with omicron as compared to the other mentioned variants may explain the reduction in the number of cases of anosmia in individuals with COVID-19. These results are in line with a very recent study that suggests a transition in cellular tropism from OE to the respiratory epithelium with omicron as compared to the WT and delta SARS-CoV-2 in the hamster model [83]. However, further research is required to fully understand how changes in the SARS-CoV-2 spike proteins between the emerging variants alter the viral tropism in the OM.

Since the start of the pandemic, several mechanisms have been hypothesized that are potentially linked to increased susceptibility of SARS-CoV-2 infection in individuals affected by AD (reviewed in [34]). Our results demonstrate that the SARS-CoV-2 virus infects equally OM-ALI cells of both cognitively healthy individuals and those affected by AD. There were no differences observed in terms of the infectability of the OM cells from control and AD individuals or the increase in the viral titer over the infection period. Therefore, our results provide crucial evidence suggesting that underlying AD pathology does not make the OM-ALI cells more vulnerable to infection. Recent research has revealed that individuals suffering from AD have higher levels of ACE-2 protein in the hippocampal region of the brain, as compared to healthy individuals. The elevated levels of ACE-2 in the brain have been linked to an augmented risk of SARS-CoV-2 neurotropism [84]. Apart from its role in facilitating viral entry into the cell, ACE-2 may also have a protective effect against the development and progression of AD by modulating the production and aggregation of Aβ, as suggested by a study in transgenic AD mice [85]. Our study conducted on OM did not reveal changes in the cellular expression of ACE-2 in AD cells, which could explain the similar infection patterns observed in both control and AD OM-ALI cells. Interestingly, we did observe that certain COVID-19-associated genes, including *CTSL* and *NRP-1*, were significantly downregulated specifically in the AD cells following infection. This supports the idea that even though healthy and AD cells are infected in a similar manner, the cellular responses to the virus may vary.

Even though the susceptibility to the infection was similar in both AD and control OM-ALI cells, the transcriptomic analyses revealed significant differential expression of genes following SARS-CoV-2 infection. In general, the data show that the overall transcriptional footprint of WT SARS-CoV-2 infection is distinct in cognitively healthy control cells in comparison to AD cells, given that 1971 DEGs were observed between SARS-CoV-2 infected control and AD OM-ALI cells. This suggests that although the virus infects the cells in the same way, the response to the infection may differ in individuals with AD, which could potentiate and intensify COVID-19-associated outcomes. It has been reported that an effective antiviral response contributes to viral clearance and improves clinical outcomes [86, 87]. However, in individuals with underlying AD, inflammation and impaired immune function can increase the risk of severe disease outcomes [88]. Interestingly in this study, SARS-CoV-2 infection in the cognitively healthy control cells shows a robust response of antiviral immune response genes and interferon-stimulated genes, which was not observed in infected cells of AD individuals. Even though the AD OM-ALI cultures without viral infection show basal enrichment of innate immune response genes and interferon-stimulated genes (Additional file 3: Fig. S3, Additional file 4: Fig.  S4), IPA analysis of the comparison between DEGs in SARS-CoV-2 infected cells from AD individuals and cognitively healthy controls indicated downregulation of *IFN-γ* (interferon-gamma) and *TNF* in the AD OM-ALI cells. In fact, innate immunity deficits, and specifically type 1 interferon signaling perturbations are often observed in AD in various cell types [89–92]. This suggests that like other cell types, the cells of the OM of individuals with AD are impaired in immune responses.

Another study suggested that in moderate-to-severe cases of SARS-CoV-2 infection, insufficient activation of interferon-mediated antiviral immune responses leads to a failure to limit viral replication in a timely manner [93]. Therefore, it is plausible that the existing activation of immune responses present in non-infected AD cells could cause desensitization or milder alterations in the respective genes after infection with SARS-CoV-2. Moreover, dampened antiviral immune and inflammatory response during early convalescence could potentially be inadequate and delay viral clearance. Recent evidence also suggested that persistent viral reservoirs and the continued SARS-CoV-2 specific immune responses during late convalescence may result in uncontrolled inflammation, causing long-lasting adverse outcomes including neurological perturbations [94]. Additionally, reactive oxygen species (ROS) are known to escalate neuroinflammation and lead to excessive production of Aβ, thereby contributing to the development and progression of AD. Notably, the current study found that infected AD cells have elevated oxidative stress as compared to infected controls. It is suggested from recent evidence that the activity of innate immunity is heavily influenced by oxidative stress, which has been identified as a significant contributor to the pathogenesis of COVID-19, due to its perpetuation of the cytokine storm cycles reported by recent data [95]. On the other hand, AD is also associated with multiple etiologies and pathophysiologic mechanisms, and oxidative stress appears to be a major part of the pathophysiologic process [96]. It is reasonable to speculate that elevated oxidative stress with persistent viral reservoirs and dysfunctional inflammatory response could potentially be linked to the worsening of existing AD pathology and progression of AD. However, further evidence is necessary to fully comprehend the long-term consequences of the infection in individuals with AD.

Loss of the sense of smell is a common attribute among many SARS-CoV-2-infected individuals and in non-infected individuals with AD. *UGTA2A* has been known to be expressed in the sustentacular cells of the OE [68, 79] and was recently implicated as a common risk gene among individuals with COVID-19-induced anosmia [59]. Our data showed upregulation of *UGTA2A* (log2fc 4.08, Padj 0.001) in mock AD cells as compared to mock control cells, suggesting an increased risk of loss of smell in AD individuals as compared to healthy controls. In addition to this, our study suggested a significant reduction in the ciliary cells after infection of the AD OM-ALI cells, as compared to infection in healthy cells. Extensive OE damage, due to loss of cells after infection and ciliary desquamation, has been reported for SARS-CoV-2 infection in the OM of mice, and hamsters [15, 75, 78]. Similarly, ciliary loss has also been indicated in the nasal and respiratory epithelium as well after infection with SARS-CoV-2 [97, 98]. Furthermore, bulk transcriptomic data from the SARS-CoV-2 infected OM-ALI cells from AD individuals show significant downregulation of olfactory receptor (OR) family genes *OR4M1, OR2T11,* and *OR4N2* after SARS-CoV-2 infection. It is important here to note that although we did not detect neurons in the OM-ALI cultures, downregulation of the OR genes was observed specifically in SARS-CoV-2 infected AD cells. While the ORs are associated primarily with the sense of smell and primarily expressed by the OSNs, recent studies have suggested that they may play a role in other biological processes in the body [99]. These receptors have been found in non-olfactory tissues such as the gut, kidney, and sperm, however, their functions beyond odorant detection have not been fully elucidated [100]. Interestingly, alterations in ORs have also been reported in the brain and implicated in neurodegenerative diseases [101]. For example, OR4M1 stimulation in mouse primary cortico-hippocampal neurons protects against abnormal tau processing [102], processing that is implicated in AD pathology. However, in this study, downregulation of the OR receptor genes after SARS-CoV-2 infection of the AD OM-ALI could possibly be attributed to hindrance in the perception of a smell. These alterations in OM cells could indicate that the presence of underlying AD may increase the risk of SARS-CoV-2 infection-associated loss of smell.

## Conclusions

In conclusion, this study introduced a novel physiologically relevant patient-derived cell model which can facilitate an improved understanding of COVID-19 pathogenesis and allow evaluation of vulnerability and risk associated with pre-existing AD in COVID-19 patients. Additionally, our model offers a pertinent preclinical platform to rapidly evaluate potential drugs and vaccines against COVID-19 and other pathogens that may emerge in the future. However, it is important to note here that our study has some limitations. First, we acknowledge that SARS-CoV-2-associated loss of smell and the infection pathogenesis in the OE can be affected by host genetics, age, ethnicity, and geographical location [103–105], however, this study was performed on primary cells derived from OM biopsies taken from AD patient and cognitively healthy individuals. Furthermore, the number of OM biopsies available for this study was restricted due to the limited availability of donors over the study duration. However, given the similar responses of the OM cells from all donors of the same disease status, we believe these results to be representative of virus-induced alterations in these cells. Second, to our knowledge, this study presents the first efforts to explore the interaction of existing AD pathology and SARS-CoV-2 infection of human-derived OM. Given that AD is a complex disease with several lifestyle and genetic factors implicated in the risk of development and progression of this disease, further studies should aim to assess the impact of confounding factors on cellular responses. Third, it is important to highlight that the scope of the study is limited to the evaluation of the effects of SARS-CoV-2 on the non-neural epithelial cell fraction of the OM cells. Although current literature suggests that direct infection of the OSNs is less probable and that infection-related effects in epithelial cells lead to detrimental effects on the neurons, this was not addressed in the current study. Research on the crosstalk between neural and non-neural cells upon infection should be a topic of further investigation given that the trans-olfactory route may serve as a potential entry route for several pathogens and environmental agents to the brain. Such studies will increase the physiological relevance and complexity to better recapitulate in vivo conditions and enable the investigation of the crosstalk at the nose–brain axis upon exposure to SARS-CoV-2 and other pathogens. Finally, this study did not address the effects of other respiratory viruses or damage-associated molecular pattern (DAMP)-induced induction of inflammation. These topics warrant further studies in the future. In conclusion, this study introduced a novel physiologically relevant patient-derived cell model to facilitate an improved understanding of COVID-19 pathogenesis and allow evaluation of vulnerability and risk associated with pre-existing AD in COVID-19 patients. Additionally, our model offers a pertinent preclinical platform to rapidly evaluate potential drugs and vaccines against COVID-19 and other pathogens that may emerge in the future.

### Supplementary Information

**Additional file 1.** Immunohistochemical staining (single channel images) of OM-ALI cultures from cognitively healthy controls for a) zonula occludens-1 (ZO1) (tight junction marker); b) acetylated tubulin (ciliary marker); c) MUC5AC (mucinproducing cells); d) Cytokeratin 18 (CK-18) (sustentacular cells); e) co-staining of nucleocapsid protein (NP) (SARS-CoV-2 infection marker) with acetylated tubulin; co-staining of nucleocapsid protein (NP) with MUC5AC. Slides were imaged on 10x objective; Scale bar 100μm.

**Additional file 2.** Significantly enriched Panther pathways at 48 h post-infection in AD cells compared to control cells. (a) ADassociated pathways are significantly enriched in mock AD cells (Control Mock vs AD Mock). (b) Infection with WT SARS-CoV-2 causes enrichment in AD-associated pathways (Control Infected vs AD Infected). (c) Numbers of genes enriched in AD-associated pathways after infection of control and AD cells.

**Additional file 3.** Changes in expression of genes involved in interferon-stimulated genes at 48 h post-infection with SARS-CoV-2. (a) Heatmap comparing mock control and infected OM-ALI cells. (b) Heatmap comparing AD mock and AD-infected OM-ALI cells.

**Additional file 4.** Changes in expression of genes involved in innate immune response at 48 h post-infection with SARS-CoV-2. (a) Heatmap comparing healthy mock and healthy infected cells. (b) Heatmap comparing AD mock and AD infected cells.

**Additional file 5: Table S1**. Significant differentially expressed genes between control mock and AD mock OM-ALI cells. **Table S2**. Significant differentially expressed genes between control mock and control-infected OM-ALI cells. **Table S3**. Significant differentially expressed genes between AD mock and AD-infected OM-ALI cells. **Table S4**. Significant differentially expressed genes between control-infected and AD-infected OM-ALI cells. **Table S5**. Significant ingenuity canonical pathways that are found from analysis of significant differentially expressed genes between control mock and AD mock. **Table S6**. Top 20 upstream regulators among significant differentially expressed genes between control mock and AD mock OM-ALI cells. **Table S7**. Significant ingenuity canonical pathways that are found from analysis of significant differentially expressed genes between control mock and control. **Table S8**. Significant ingenuity canonical pathways that are found from analysis of significant differentially expressed genes between AD mock and AD infected. **Table S9**. Top 20 upstream regulators among significant differentially expressed genes between control mock and control-infected OM-ALI cells. Table S10 Top 20 upstream regulators among significant differentially expressed genes between AD mock and AD-infected OM-ALI cells. **Table S11**: Significant ingenuity canonical pathways that are found from analysis of significant differentially expressed genes between control infected and AD. **Table S12.** Top 20 upstream regulators among significant differentially expressed genes between control-infected and AD-infected OM-ALI cells.

## Data Availability

The data presented in this study are available upon reasonable request from the corresponding author. RNA sequencing data will be available from the European Genome–phenome Archive (EGA, https://ega-archive.org/) under the The Data Access Committee for Human Olfactory Mucosa Cells (DAC_HOM) at UEF (EGAC00001002527).

## Abbreviations

COVID-19: Coronavirus disease of 2019
AD: Alzheimer’s disease
OM: Olfactory mucosa
SARS-CoV-2: Severe acute respiratory syndrome coronavirus 2
ALI: Air–liquid interface
NP: Nucleocapsid protein
OSNs: Olfactory sensory neurons
LP: Lamina propria
OE: Olfactory epithelium
D-PBS: Dulbecco’s phosphate buffered saline
HBSS: Hank’s balanced salt solution
OM-ALI: Olfactory mucosa cell at air–liquid interface
WT: Wild type
TEER: Trans-epithelial electrical resistance
hpi: Hour post-infection
DEGs: Differentially expressed genes
PFA: Paraformaldehyde
mRNA: Messenger RNA
PFU: Plaque forming unit
BSA: Bovine serum albumin
CNS: Central nervous system
BSL: Biosafety level 2
IPA: Ingenuity pathway analysis
ACE-2: Angiotensin-converting enzyme 2
TMPRSS2: Transmembrane protease, serine 2
NRP-1: Neuropilin-1
CTSB: Cathepsin B
CTSL: Cathepsin L
BSG: Basigin
Furin: Furin
UGT2A2: UDP Glucuronosyltransferase Family 2 Member A2
CXCL10: C–X–C motif chemokine ligand 10
OASL: 2ʹ-5ʹ-Oligoadenylate synthetase like
RSAD2: Radical *S*-adenosyl methionine domain containing 2
IFIT1: Interferon induced protein with tetratricopeptide repeats 1
PTGS2: Prostaglandin-endoperoxide synthase 2
elF2: Eukaryotic translation initiation factor 2
APOE: Apolipoprotein E
OR: Olfactory receptor
MUC5AC: Mucin 5AC
TNF: Tumor necrosis factor
