# Supplementary figures and images for: Human-derived air–liquid interface cultures decipher Alzheimer’s disease–SARS-CoV-2 crosstalk in the olfactory mucosa
Source: J Neuroinflammation. 2023 Dec 14;20:299. doi: 10.1186/s12974-023-02979-4 (PMC10722731; doi:10.1186/s12974-023-02979-4)

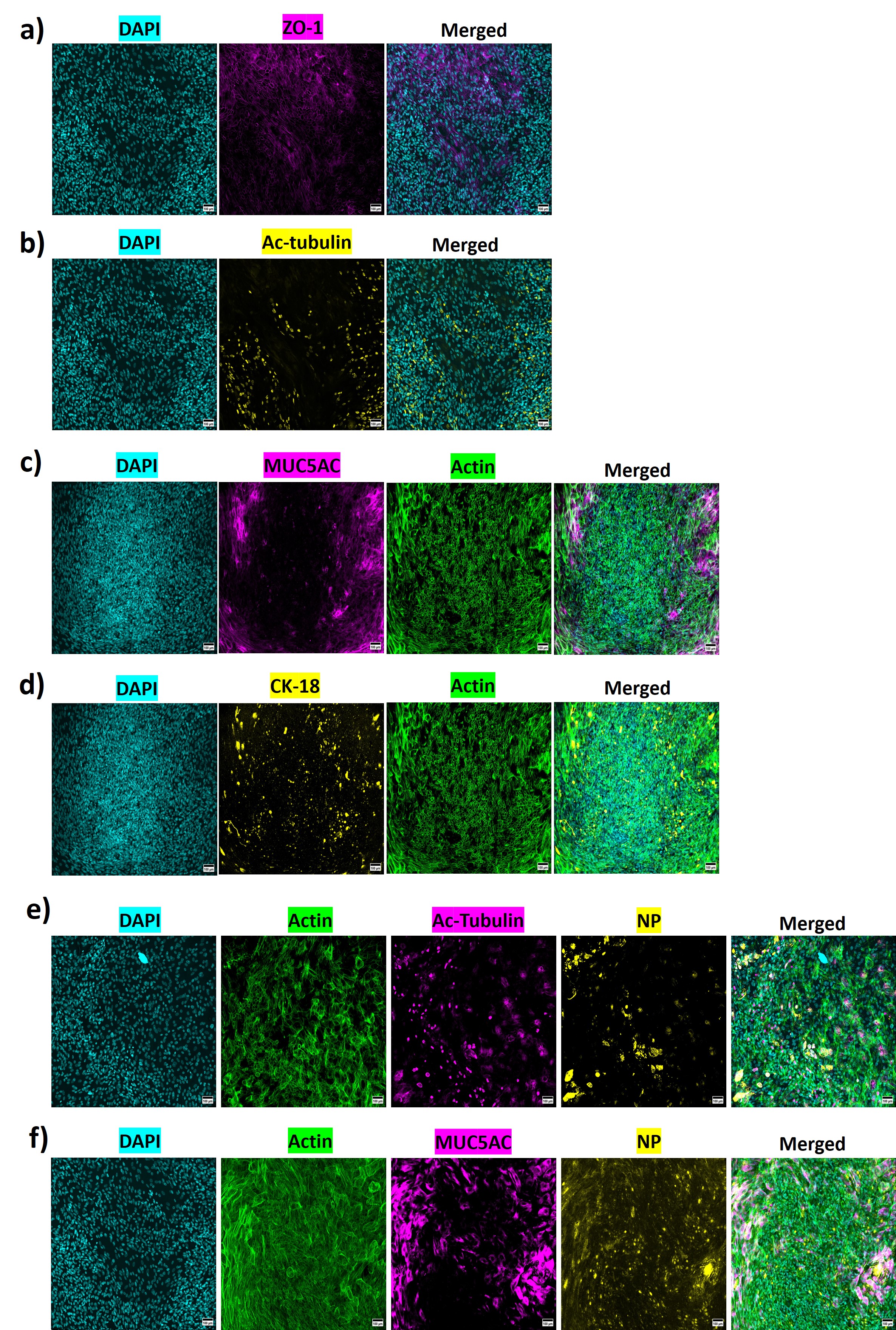

Supplement: Supplementary file 1 — Additional file 1. Immunohistochemical staining (single channel images) of OM-ALI cultures from cognitively healthy controls for a) zonula occludens-1 (ZO1) (tight junction marker); b) acetylated tubulin (ciliary marker); c) MUC5AC (mucinproducing cells); d) Cytokeratin 18 (CK-18) (sustentacular cells); e) co-staining of nucleocapsid protein (NP) (SARS-CoV-2 infection marker) with acetylated tubulin; co-staining of nucleocapsid protein (NP) with MUC5AC. Slides were imaged on 10x objective; Scale bar 100μm. [file 12974_2023_2979_MOESM1_ESM.jpg]

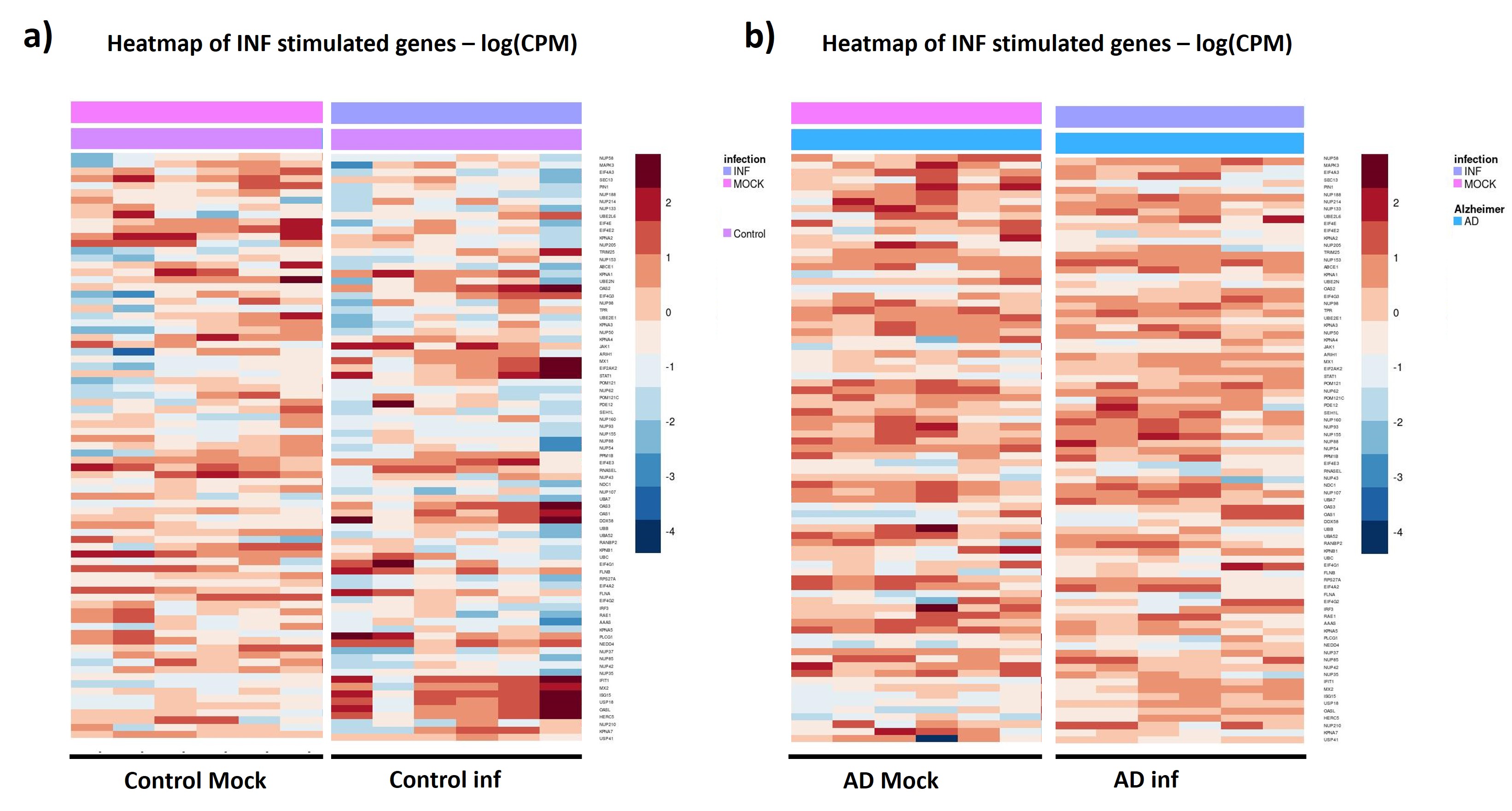

Supplement: Supplementary file 3 — Additional file 3. Changes in expression of genes involved in interferon-stimulated genes at 48 h post-infection with SARS-CoV-2. (a) Heatmap comparing mock control and infected OM-ALI cells. (b) Heatmap comparing AD mock and AD-infected OM-ALI cells. [file 12974_2023_2979_MOESM3_ESM.jpg]

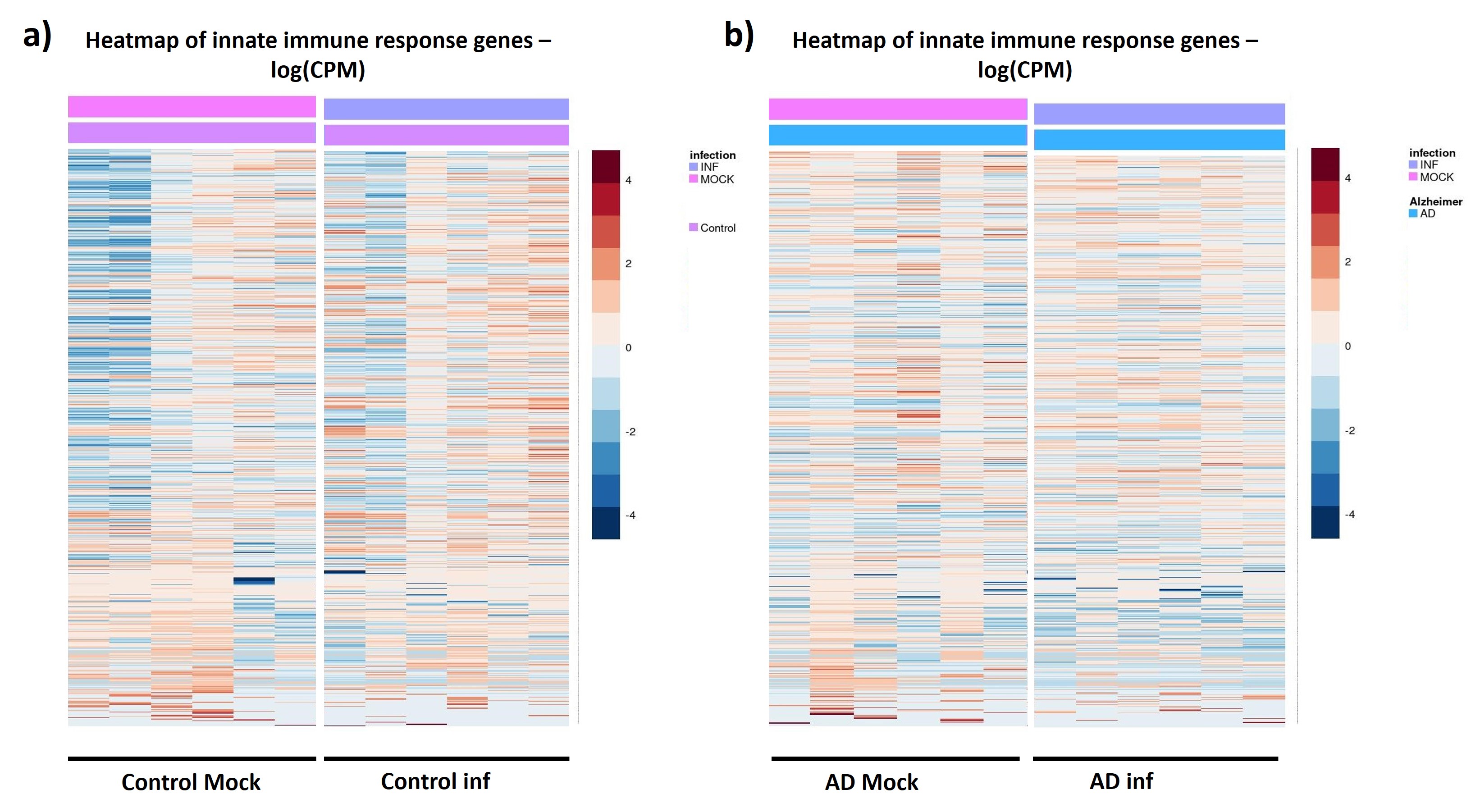

Supplement: Supplementary file 4 — Additional file 4. Changes in expression of genes involved in innate immune response at 48 h post-infection with SARS-CoV-2. (a) Heatmap comparing healthy mock and healthy infected cells. (b) Heatmap comparing AD mock and AD infected cells. [file 12974_2023_2979_MOESM4_ESM.jpg]
